# Pro- and anti-inflammatory cytokines and growth factors in patients undergoing *in vitro* fertilization procedure treated with prednisone

**DOI:** 10.3389/fimmu.2023.1250488

**Published:** 2023-09-06

**Authors:** Karolina Piekarska, Marta Dratwa, Paweł Radwan, Michał Radwan, Katarzyna Bogunia-Kubik, Izabela Nowak

**Affiliations:** ^1^ Laboratory of Immunogenetics and Tissue Immunology, Department of Clinical Immunology, Hirszfeld Institute of Immunology and Experimental Therapy, Polish Academy of Sciences, Wrocław, Poland; ^2^ Laboratory of Clinical Immunogenetics and Pharmacogenetics, Department of Clinical Immunology, Hirszfeld Institute of Immunology and Experimental Therapy, Polish Academy of Sciences, Wrocław, Poland; ^3^ Department of Reproductive Medicine, Gameta Hospital, Rzgów, Poland; ^4^ Faculty of Health Sciences, The Mazovian Academy in Plock, Płock, Poland

**Keywords:** *in vitro* fertilization embryo transfer, recurrent implantation failure, cytokines, proinflammatory, anti-inflammatory

## Abstract

Embryo implantation is a key moment in pregnancy. Abnormal production of pro- and anti-inflammatory cytokines, their receptors and other immune factors may result in embryo implantation failure and pregnancy loss. The aim of this study was to determine the profile of selected pro- and anti-inflammatory factors in the blood plasma of patients undergoing *in vitro* fertilization (IVF) and control women who achieved pregnancy after natural conception. The examined patients were administered steroid prednisone. We present results concern the plasma levels of IFN-ɣ, BDNF, LIF, VEGF-A, sTNFR1 and IL-10. We found that IVF patients receiving steroids differed significantly from patients who were not administered such treatment in terms of IFN-γ and IL-10 levels. Moreover, IVF patients differed in secretion of all tested factors with the fertile controls. Our results indicated that women who secrete at least 1409 pg/ml of sTNFR1 have a chance to become pregnant naturally and give birth to a child, while patients after IVF must achieve a concentration of 962.3 pg/ml sTNFR1 in blood plasma for successful pregnancy. In addition, IVF patients secreting VEGF-A above 43.28 pg/ml have a greater risk of miscarriage or a failed transfer in comparison to women secreting below this value. In conclusion, fertile women present a different profile of pro- and anti-inflammatory cytokines, and growth factors compared to patients with recurrent implantation failure (RIF).

## 1 Introduction

Embryo implantation is a crucial process in the development and maintenance of a pregnancy. It begins with blastocyst adhesion to the surface of the uterus. Trophoblast cells, derived from the trophectoderm, form columns of anchoring villi and differentiate into syncytiotrophoblast and extravillous trophoblast (EVT). Furthermore, extravillous trophoblast migrate to the maternal uterus and develop into a placenta (1, 2). Implantation failure may occur during early stages of embryo development. Inability to naturally conceive is the primary reason behind couples using assisted reproductive techniques (ART). Despite significant advances in ART, such as *in vitro* fertilization (IVF), women undergoing these procedures experience recurrent implantation failure (RIF) (3, 4). The exact definition of RIF is difficult to determine, but most clinicians define RIF as a failure to achieve a pregnancy after three subsequent IVF cycles in which four good-quality embryos are transferred into women under the age 40 years (4–6). The pathogenesis of recurrent embryo implantation failure is ambiguous. There are many risk factors that may contribute to RIF, with the primary causes being maternal, male or embryo factors (4). During pregnancy the immune system has the dual purpose to protect the mother and the developing fetus against pathogens while also allowing tolerance for the semi-allogenic fetus, which expresses antigens from both the mother and the father. During trophoblast invasion and at birth, the decidua is dominated by pro-inflammatory factors, while anti-inflammatory factors are dominant in the second and third trimesters of pregnancy to facilitate fetal growth (7, 8).

Cytokines mediate signals between the endometrium during embryo implantation and throughout pregnancy. They may promote trophoblast invasion and placental formation. Maintaining the right balance between pro-inflammatory and anti-inflammatory cytokines is essential for pregnancy development and disorders or infections can alter cytokine expression (9). Cytokines can be secreted by endometrial cells and by immune system cells recruited into the uterus to the site of implantation. They are mostly uterine natural killer cells, macrophages, and dendritic cells (10–14). T cells also exhibit complex and variable cytokine production. Th1 cells are mainly involved in the elimination of cells infected with viruses and intracellular pathogens. Th1 cytokines (considered as pro-inflammatory) include interferon-ɣ (IFN-ɣ), tumor necrosis factor-α (TNF-α) and the interleukins: IL-1, IL-2, IL-12, IL-15, IL-18 (15, 16). Th1 cytokines predominate during the early stages of implantation and are thought to promote trophoblast invasion. Higher levels of TNF-α before embryo transfer have been shown to be associated with clinical pregnancy in RIF patients, emphasizing that pro-inflammatory factors are needed for embryo implantation (17, 18). However, studies concerning TNF-α are ambiguous because a meta-analysis performed by Zhang et al. showed an increased level of this cytokine in patients with recurrent spontaneous miscarriage in comparison to the controls (19). TNF-α works by binding to the TNF-α receptors (TNFR1 and TNFR2). Moreover, TNF receptors (TNFR1 and also TNFR2) expressed on trophoblasts may play a role in regulating development of fetus in pregnancy (20–22). After embryo implantation, in the second trimester of pregnancy, a noticeable shift occurs and the Th2 (anti-inflammatory) cytokines begin to predominate in the uterus, these include: IL-4, IL-5, IL-10, IL-13 and granulocyte-macrophage colony-stimulating factor (GM-CSF). Th2 cytokines are responsible for fetal growth and maintenance of the pregnancy. The prevalence of the Th1 response over Th2 may be associated with various complications of pregnancy, for example recurrent miscarriages, pre-eclampsia and embryo implantation failure after *in vitro* fertilization (9, 23–25). In the third trimester of pregnancy, the Th1 inflammatory response returns. Of great importance in this phase is the infiltration of immune cells into the myometrium, which supports uterine contractions, labor and placental separation (10, 26).

Cytokines may have an effect on endometrial decidualization. This is the process of endometrial stromal cell differentiation into specialized decidual cells that create the lining for the implanting embryo. Cytokines promote blood vessel formation and increase blood flow in the decidua. Uterine NK cells (uNK) are capable of secreting angiogenic factors such as vascular endothelial growth factor (VEGF) and placental growth factor (PlGF) (27). PlGF may have both a pro-angiogenic or anti-angiogenic effect, therefore effective decidualization requires the appropriate expression levels of this factor (28). VEGF can regulate the proliferation and survival of endothelial cells and increases the permeability of blood vessels. Therefore, VEGF supports the differentiation of trophoblast cells and is responsible for angiogenesis in the decidua (28, 29). It is reported that VEGF-A is the most important and potent factor in angiogenesis (30). Brain-derived neurotrophic factor (BDNF), which belongs to the neurotrophin family, is involved in the proliferation, differentiation and survival of neuronal cells. BDNF also plays a role in placental development, differentiation and proliferation of trophoblasts (31). Reduced circulating levels of BDNF in the sera of women undergoing IVF were probably associated with adverse peri- and/or post-implantation events and subsequent pregnancy failure (32).

A significant role in embryo implantation may also be played by leukemia inhibitory factor (LIF). It is expressed in the endometrium at the time of implantation by uNK cells and its receptor is located both in the endometrium and on the trophoblast (33, 34). By regulating prostaglandin synthesis, LIF may affect embryo implantation and decidualization. Studies have shown that LIF can enhance the expression of human leukocyte antigen (HLA-G) in choriocarcinoma cell line JEG3, used as a model for trophoblasts (35). This induction occurred in the presence of endoplasmic reticulum aminopeptidase-1 (ERAP1), which is involved in HLA class I antigenic peptide presentation through the trimming peptides to their optimal length.

Pro-inflammatory and anti-inflammatory cytokines and cytokine receptor profiles may change in the plasma of patients before and after the *in vitro* fertilization procedure. Abnormal production of cytokines and other immune factors may result in embryo implantation failure and pregnancy loss. Measurement of the level of these factors in the plasma of patients undergoing IVF before and after the procedure may potentially be useful and have prognostic value in the diagnosis of RIF. Therefore, the aim of this study was to determine the immunological profile of pro- and anti-inflammatory cytokines in the blood plasma of patients undergoing *in vitro* fertilization before and after embryo transfer and control women who achieved pregnancy after natural conception. We investigated plasma levels of IFN-ɣ, GM-CSF, TNF-α, PlGF, BDNF, LIF, VEGF-A, VEGF-D, TNFR1 and interleukins: IL-10, IL-4, IL-6. The patients under examination were administered the steroid prednisone, which prompted our novel investigation into the influence of steroids on the secretion of specific cytokines.

## 2 Materials and methods

### 2. 1 Study design

One hundred eighty-seven patients were involved in this study (age range: 22-46), all underwent *in vitro* fertilization embryo transfer. Patients were enrolled into the study in years 2015 to 2020. They were qualified at the Gameta Assisted Reproduction Clinic in Rzgów, a centre certified by the European Society for Human Reproduction and Embryology (ESHRE ART Centre Certification for good clinical practice) and at the Department of Surgical, Endoscopic and Oncologic Gynecology, Department of Gynecology and Gynecologic Oncology, Polish Mothers’ Memorial Hospital–Research Institute. Patients underwent an average 3 unsuccessful transfers and each had 3 good quality embryos transferred. In 67 patients (35.82%) IVF was indicated due to male only fertility factors, while female factors accounted for 45 patients (24.06%). Twenty-eight patients (14.97%) were infertile due to male and female factors, while idiopathic infertility was found in 47 patients (25.13%).

Eighty-seven percent of all patients (164 women) were administered prednisone. In the standard IVF procedure, patients were instructed to take 5 mg of prednisone on a routine basis (Encorton, Adamed, Poland) once a day in the morning orally, starting from the day of embryo transfer (ET/frozen ET). Patients with RIF received higher doses of prednisone (10–20 mg) for 2–3 weeks before ET and up to 8 weeks if pregnancy developed after transfer. Among those patients taking prednisone, 85 women achieved clinical pregnancy, 42 women experienced a miscarriage and in the case of 37 patients the embryo transfer resulted in a lack of pregnancy.

In order to supplement the luteal phase, all patients were intravaginally administered 2 × 200 mg micronized progesterone (Luteina, Adamed, Poland) and oral dydrogesterone 3 × 10 mg (Duphaston, Solvay Pharmaceuticals, Netherlands) until 12 weeks of gestation. Detailed information on the preparation of patients for IVF [ovarian stimulation, fertilization procedure, assessment of endometrial thickness for initiation progesterone supplementation and frozen ET (FET)] has been described in an earlier by Nowak et al. (36).

The fertile control group was recruited from the Institute of Immunology and Experimental Therapy of the Polish Academy of Sciences in the years 2018-2020. This group consisted of 67 healthy women (age range: 19–68) with at least 1 healthy-born child after natural conception and no history of miscarriage or endocrinological or immunological diseases, except five women, who had hypothyroidism. Among fertile control, we had samples from 27 pregnant women from natural conception. The gestational age of these women was from 6 to 37 weeks at the moment of blood donation. None of the fertile control patients were administered any steroid treatment.

All tested patients were of Polish origin. Experiments were carried out after obtaining the approval of the Ethics Committee of the Polish Mothers’ Memorial Hospital–Research Institute in Łódź (No: 25/2019). The necessary consent was obtained from all individual participants. IVF patients differed significantly in mean age from fertile women (p = 0.0414). The clinical characteristics of patients and fertile women are described in **Table 1**.

**Table 1:** Characteristics of patients and the fertile control groups.

Parameter		IVF	Fertile control	Fertile pregnant control
Number of women		N = 187	N = 40	N = 27
Age of women	Mean ± SD	32.99 ± 4.15	32.93 ± 6.03	31.69 ± 5.13
	Range	22-46	22-68	19-42
Indications for IVF-ET (%)	Male only factor	67 (35.82)	–	–
	Female only factor	45 (24.06)	–	–
	Both factors	28 (14.97)	–	–
	Unknown factor	47 (25.13)	–	–
Number of IVF-ET in all cycles	Mean ± SD	3.08 ± 0.15	–	–
	Range	1-11	–	–
Number of embryos in all cycles	Mean ± SD	3.39 ± 2.38	–	–
	Range	1-13	–	–

### 2.2 Measurement of pro- and anti-inflammatory cytokines and growth factors levels in plasma

Plasma samples from patients were taken before and after IVF-ET [at the moment of testing for beta-subunit of human chorionic gonadotropin (β-hCG)] between 11 and 15 days following the ET date) and also from fertile women. Samples were stored at -80 °C until the time of assay. Plasma levels of GM-CSF, IFN-ɣ, IL-10, IL-4, TNF-α (pg/ml) were detected by Human ProcartaPlex High Sensitivity assay (Thermo Fisher Scientific, USA) using the Luminex 200 system (Luminex Corp., USA). Concentrations of BDNF, IL-6, PlGF, LIF, TNF-R1, VEGF-A, VEGF-D (pg/ml) were measured by the commercial Human ProcartaPlex assay (Thermo Fisher Scientific, USA) also using the Luminex 200 system (Luminex Corp., USA). All measurements were performed following the protocol of the manufacturer. Luminex technology is based on colored magnetic microspheres coated with antibodies that capture specific analytes in a multiplex ELISA. The median fluorescence intensity was compared to the standard curve to calculate the analyte concentration in pg/mL with xPonent 4.2 software. Standard curves for each analyte were generated using the premixed lyophilized standards provided in the kits. Serial 4-fold dilutions of the standards were run to generate a 7-standard concentration set, and the diluent alone was used as a blank. The analyte concentrations in the samples were determined from the standard curve using a 5-parameter logistic fit curve to transform the mean fluorescence intensities into concentrations. There is a different detection range for each analyte. Standard curves measured the concentration were: for IFN-ɣ from 1.44 to 5900 pg/ml, for IL-10 from 0.19 to 765 pg/ml, for BDNF from 1.71 to 7000 pg/ml, for LIF from 11.06 to 45300 pg/ml, for TNFR1 from 158.96 to 651100 pg/ml, for VEGF-A from 5.49 to 22500 pg/ml.

### 2.3 Statistical analysis

To assess the differences in the concentration of the selected cytokines and growth factors in patient plasma before and after ET, the Mann–Whitney test was performed (GraphPad Prism 5 software). The normality of the data distributions was assessed by the D’Agostino-Pearson test. Statistical significance was considered as p value < 0.05. All parameters of statistical analyses (numbers, medians, means, standard deviation and errors, min, max and 25-75% percentiles) are part of **Supplementary Tables 1-12**. Results of the statistical analysis referring to cytokines and growth factors such as GM-CSF, IL-4, TNF-α, IL-6, PlGF and VEGF-D failed to yield any conclusions due to undetectable levels of cytokines. The median fluorescence intensities of tested analytes were not intensive enough to calculate their concentration. Therefore they were omitted from this manuscript.

## 3 Results

### 3.1 Secretion of IFN-ɣ in patients and the fertile control groups

**Figure 1:**
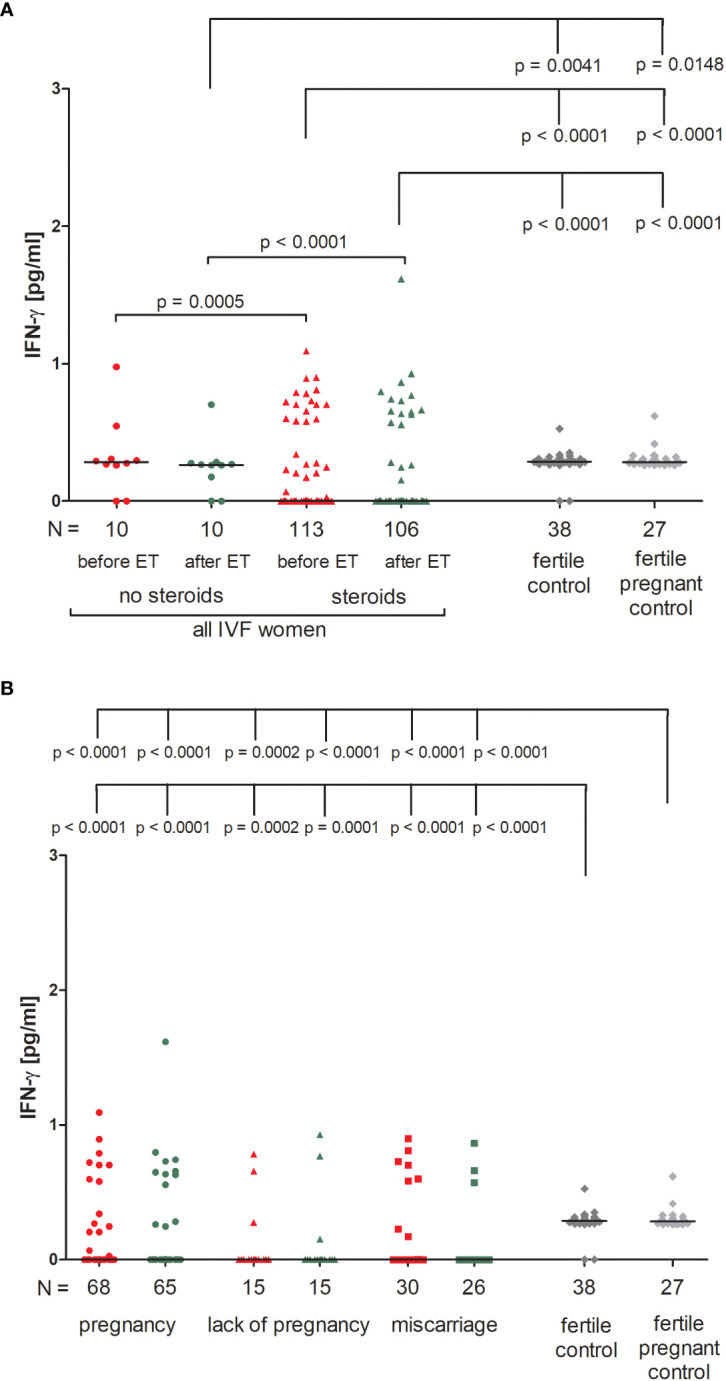
Concentration of plasma IFN-ɣ measured before and after embryo transfer in all patients and fertile controls. **(A)** Concentration of IFN-ɣ (pg/ml) measured before and after embryo transfer in patients receiving steroid or no steroid treatment and in fertile controls (without steroid treatment); **(B)** Concentration of IFN-ɣ (pg/ml) measured before and after embryo transfer in steroid treatment patients who achieved clinical pregnancy, experienced a lack of pregnancy or miscarriage and in fertile controls (without steroid treatment). Red points indicate measurement before embryo transfer; green, after embryo transfer; dark grey points, fertile control; light grey points, fertile pregnant control. *N* is the numer of women. Black horizontal lines represent medians. P values are calculated by Mann-Whitney test.

We found that IVF patients who were prescribed steroids differed significantly from patients who were not. IVF patients without steroid treatment had higher levels of IFN-ɣ before and after ET in comparison to patients receiving steroid treatment (p = 0.0005, median 0.28 vs. 0.00 pg/ml and p < 0.0001, median 0.26 vs. 0.00 pg/ml, respectively; **Figure 1A**; **Supplementary Table 1**). Fertile women who had given birth in the past and women who were pregnant at the time of blood collection differed in terms of IFN-ɣ concentrations compared to IVF patients taking prednisone before and after ET (p < 0.0001 for comparisons with IVF steroid treatment patients, median 0.29 vs. 0.00 pg/ml before and after ET, median 0.28 vs. 0.00 pg/ml before and after ET). IVF women untreated with steroid secreted less IFN-ɣ after ET than fertile women and fertile pregnant women (p = 0.0041, median 0.26 vs. 0.29 pg/ml and p = 0.0148, median 0.26 vs. 0.28 pg/ml, respectively).

IVF patients taking prednisone, the overwhelming majority, did not secrete IFN-ɣ (all medians 0.000 pg/ml), therefore we only found statistically significant differences between those patients and women from control groups (median 0.29 and 0.28 pg/ml). The concentration of IFN-ɣ measured before and after ET differed in patients who became pregnant and fertile women who had given birth in the past and pregnant control (p < 0.0001 for both patient groups before and after ET; **Figure 1B**; **Supplementary Table 2**). We observed the same differences (p < 0.0001 or p = 0.0002) in the analysis of patients who experienced a lack of pregnancy or had miscarriages and women from the control groups.

### 3.2 IL-10 secretion in IVF patients and the fertile control groups

**Figure 2:**
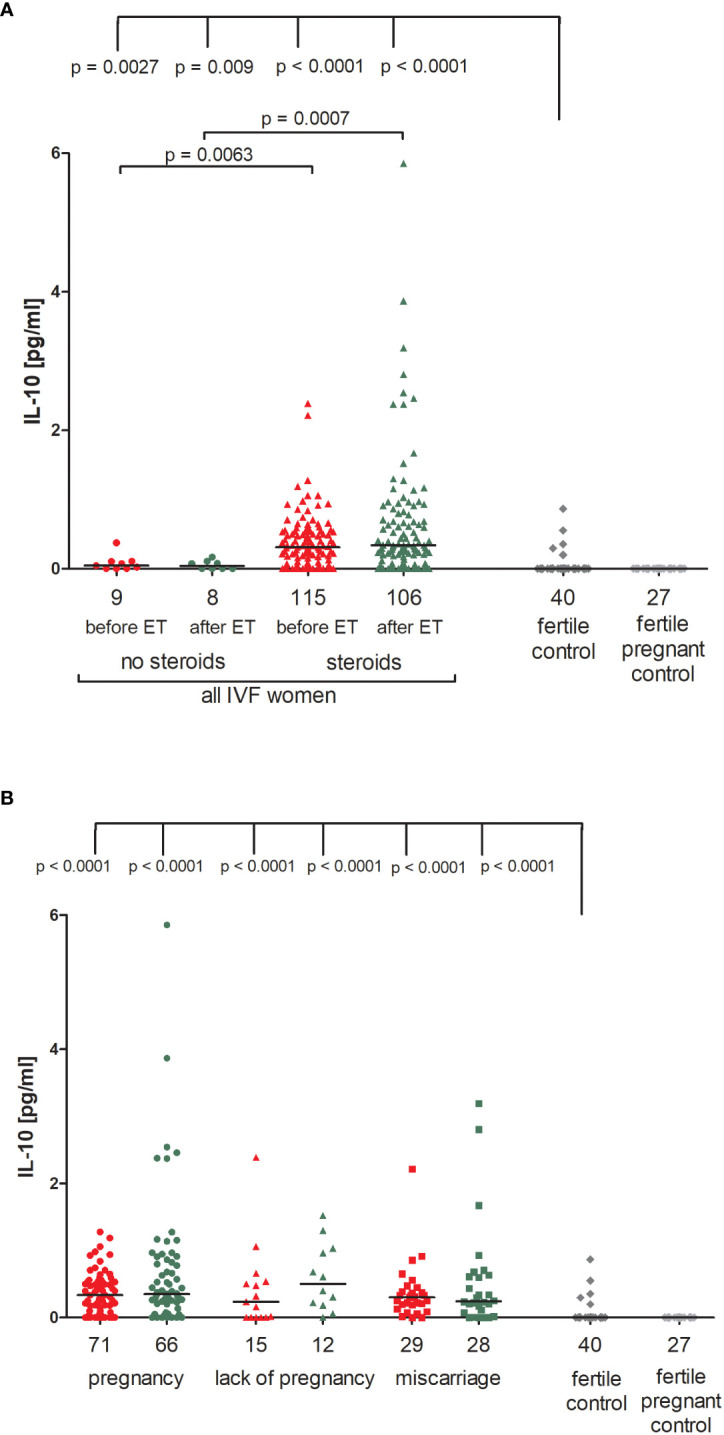
Concentration of plasma IL-10 measured before and after embryo transfer in all patients and fertile controls. **(A)** Concentration of IL-10 (pg/ml) measured before and after embryo transfer in patients receiving steroid or no steroid treatment and in fertile controls (without steroid treatment); **(B)** Concentration of IL-10 (pg/ml) measured before and after embryo transfer in steroid treatment patients who achieved clinical pregnancy, experienced a lack of pregnancy or miscarriage and in fertile controls (without steroid treatment). Red points indicate measurement before embryo transfer; green, after embryo transfer; dark grey points, fertile control; light grey points, fertile pregnant control. *N* is the numer of women. Black horizontal lines represent medians. P values are calculated by Mann-Whitney test.

We observed that patients undergoing steroid treatment secreted significantly more IL-10 into plasma than patients without steroid treatment (p = 0.0063, median 0.31 vs. 0.05 pg/ml before ET; p = 0.0007, median 0.34 vs. 0.04 pg/ml after ET; **Figure 2A**; **Supplementary Table 3**). Most fertile women did not secrete IL-10 (median 0.00 pg/ml) and they differed to IVF patients receiving steroid treatment and those without in terms of IL-10 plasma levels (for patients with steroid treatment: p = 0.0027, median 0.00 vs. 0.05 pg/ml before ET, p = 0.009, median 0.00 vs. 0.04 pg/ml after ET; for patients without steroid treatment: p < 0.0001, median 0.00 vs. 0.31 pg/ml before ET; p < 0.0001, median 0.00 vs. 0.34 pg/ml after ET).

The concentration of IL-10 measured before and after ET did not differ in IVF women using steroid and achieved pregnancy (median 0.34 pg/ml before and 0.35 pg/ml after ET) and patients who experienced a lack of pregnancy (median 0.24 pg/ml before and 0.50 pg/ml after ET) or patients who experienced a miscarriage (median 0.30 pg/ml before and 0.24 pg/ml after ET) (**Figure 2B**; **Supplementary Table 4**). However, we noticed a decreased level of IL-10 in women who conceived naturally and gave birth to a child (median 0.00 pg/ml). The difference in concentrations between the fertile controls and all these patients was p < 0.0001, both before and after embryo transfer (**Figure 2B**; **Supplementary Table 4**).

### 3.3 Secretion of BDNF in IVF patients and the fertile control groups

**Figure 3:**
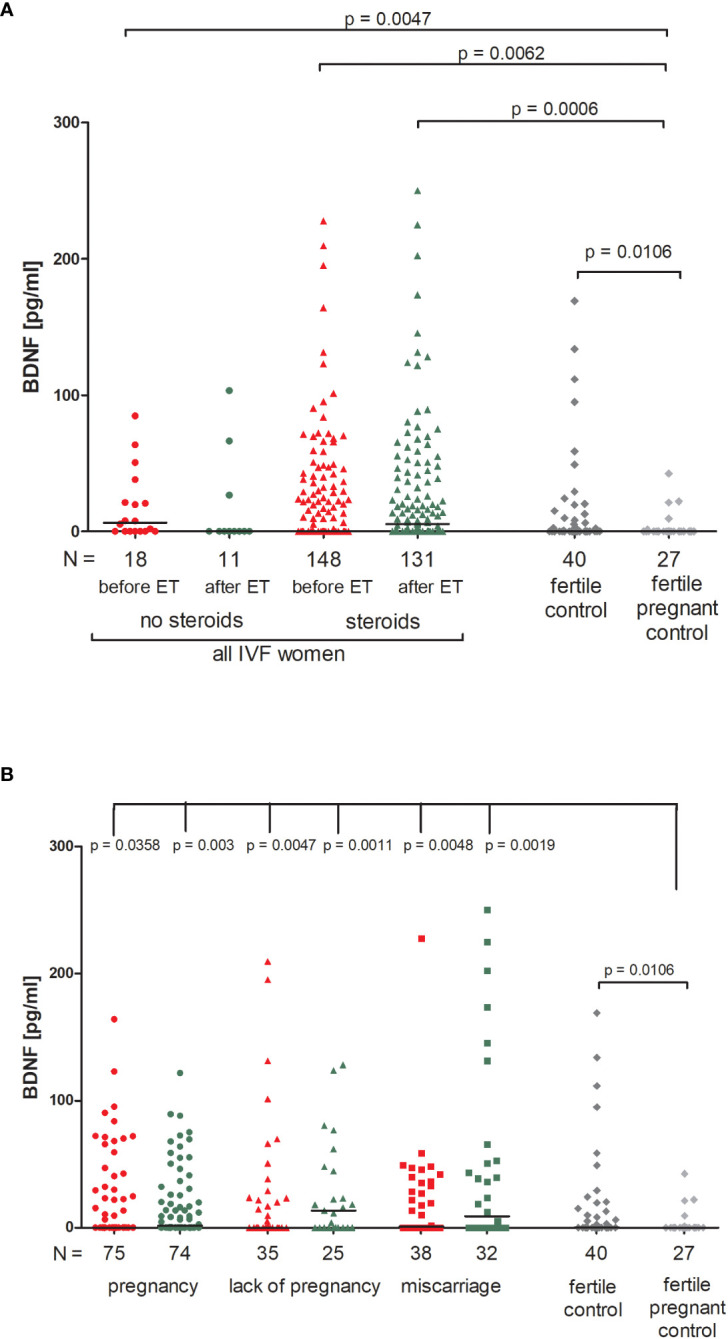
Concentration of plasma BDNF measured before and after embryo transfer in all patients and fertile controls. **(A)** Concentration of BDNF (pg/ml) measured before and after embryo transfer in patients receiving steroid or no steroid treatment and in fertile controls (without steroid treatment); **(B)** Concentration of BDNF (pg/ml) measured before and after embryo transfer in steroid treatment patients who achieved clinical pregnancy, experienced a lack of pregnancy or miscarriage and in fertile controls (without steroid treatment). Red points indicate measurement before embryo transfer; green, after embryo transfer; dark grey points, fertile control; light grey points, fertile pregnant control. *N* is the numer of women. Black horizontal lines represent medians. P values are calculated by Mann-Whitney test.

We found no significant differences between patients who received steroid treatment and patients who did not in terms of BDNF secretion into plasma (**Figure 3A**; **Supplementary Table 5**). On the other hand, fertile women who were pregnant at the moment of blood collection had the lowest level of BDNF (median 0.00 pg/ml). Therefore, this group of women differed in secretion of BDNF with patients receiving steroid treatment (p = 0.0047, median 6.52 pg/ml before ET) and patients without steroid treatment (p = 0.0062, median 0.00 pg/ml before ET; p = 0.0006, median 5.38 pg/ml after ET). Women with a current natural pregnancy also had a lower level of BDNF than women who had given birth in the past previously from natural conception (p = 0.0106, median 0.00 vs. 0.30 pg/ml).

When we divided patients receiving steroid treatment according to their pregnancy outcome after embryo transfer, we observed significant differences between all patients and the fertile pregnant controls (**Figure 3B**; **Supplementary Table 6**). The most striking difference in concentration of BDNF was observed in fertile pregnant women and patients for whom the embryo transfer did not result in a pregnancy (p = 0.0011, median 0.00 vs. 13.53 pg/ml after ET, respectively). Furthermore, the level of BDNF before ET in patients with a lack of pregnancy was higher than in the fertile pregnant controls (p = 0.0047, median 0.11 vs. 0.00 pg/ml, respectively). When embryo transfer resulted in pregnancy, we also observed statistically significant differences between patients and the fertile pregnant controls (p = 0.0358, median 0.00 before ET vs. 0.00 pg/ml, p = 0.003, median 1.63 vs. 0.00 pg/ml, respectively). Additionally, patients who experienced a miscarriage had a higher secretion of BDNF than in the fertile pregnant controls (p = 0.0048, median 0.94 before ET vs. 0.00 pg/ml; p = 0.0019, median 8.93 vs. 0.00 pg/ml).

### 3.4 LIF secretion in IVF patients and fertile groups

**Figure 4:**
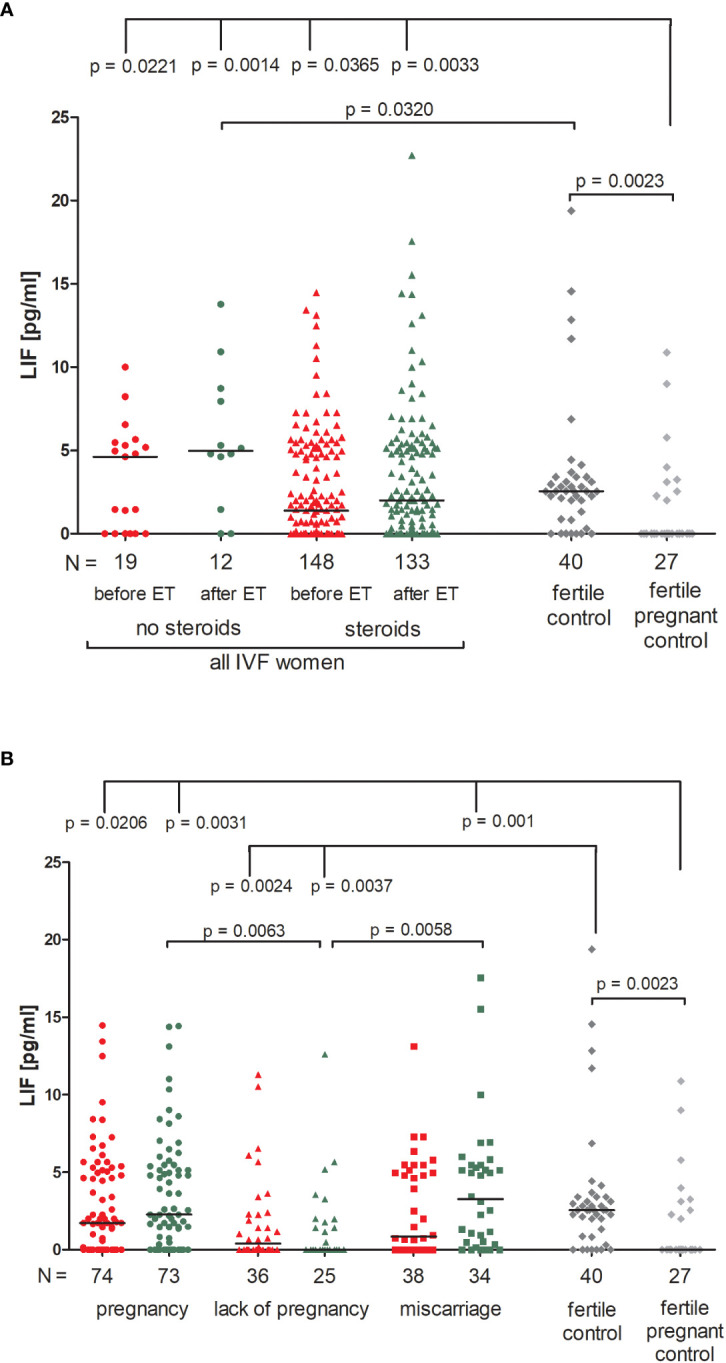
Concentration of plasma LIF measured before and after embryo transfer in all patients and fertile controls. **(A)** Concentration of LIF (pg/ml) measured before and after embryo transfer in patients receiving steroid or no steroid treatment and in fertile controls (without steroid treatment); **(B)** Concentration of LIF (pg/ml) measured before and after embryo transfer in steroid treatment patients who achieved clinical pregnancy, experienced a lack of pregnancy or miscarriage and in fertile controls (without steroids treatment). Red points mean measurement before embryo transfer; green, after embryo transfer; dark grey points, fertile control; light grey points, fertile pregnant control. *N* is the numer of women. Black horizontal lines represent medians. P values are calculated by Mann-Whitney test.

Patients who were not receiving steroid treatment had higher LIF values both before and after ET, but the differences compared to patients using prednisone were not significant (**Figure 4A**; **Supplementary Table 7**). All patients had a higher concentration of LIF than in the fertile pregnant controls (p = 0.0221, median 4.62 before ET vs. 0.00 pg/ml and p = 0.0014, median 4.94 after ET vs. 0.00 pg/ml for patients not receiving steroid treatment; p = 0.0365, median 1.38 before ET vs. 0.00 pg/ml and p = 0.0033, median 2.00 after ET vs. 0.00 pg/ml for patients receiving steroid treatment). In addition, patients with no steroid treatment had a higher level of LIF after ET in comparison to the fertile controls (p = 0.0320, median 4.96 vs. 2.55 pg/ml, respectively). We also observed different secretion levels of LIF in fertile women and fertile pregnant women (p = 0.0023, median 2.55 vs. 0.00 pg/ml, respectively; **Figure 4A**; **Supplementary Table 7**).

When we compared patients according to various pregnancy outcomes, we observed that the lowest level of LIF in plasma was found in those patients who experienced a lack of pregnancy, in particular there was a concentration decrease after embryo transfer (**Figure 4B**; **Supplementary Table 8**). Comparison between patients who experienced a lack of pregnancy and patients who achieved pregnancy after ET (p = 0.0063, median 0.00 vs. 2.27 pg/ml after ET) and also between patients who had a miscarriage (p = 0.0058, median 0.00 vs. 3.27 pg/ml after ET) was significant. P values were also significant in comparison of the LIF secretion between patients with a lack of pregnancy and fertile women (p = 0.0024, median 0.40 before ET vs. 2.55 pg/ml; p = 0.0037, median 0.00 after ET vs. 2.55 pg/ml). Moreover, patients who achieved pregnancy after ET and also patients who experienced a miscarriage had higher levels of LIF than the fertile pregnant controls (p = 0.0206, median 1.73 before ET vs. 0.00 pg/ml; p = 0.0031, median 2.27 after ET vs. 0.00 pg/ml and p = 0.001, median 3.27 after ET vs. 0.00 pg/ml; respectively; **Figure 4B**; **Supplementary Table 8**).

### 3.5 Secretion of soluble TNFR1 in IVF patients and the fertile control groups

**Figure 5:**
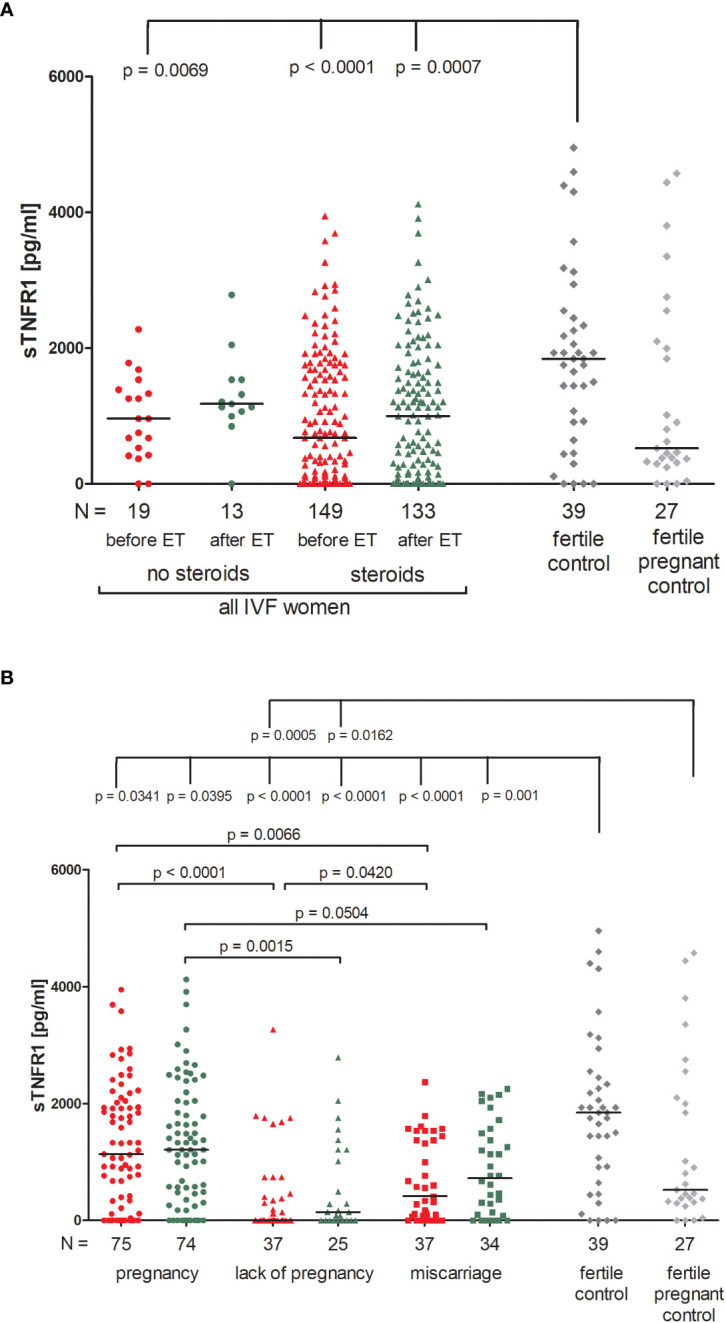
Concentration of soluble TNFR1 measured before and after embryo transfer in all patients and fertile controls. **(A)** Concentration of soluble TNFR1 (pg/ml) measured before and after embryo transfer in patients receiving steroid or no steroid treatment and in fertile controls (without steroid treatment); **(B)** Concentration of soluble TNFR1 (pg/ml) measured before and after embryo transfer in steroid treatment patients who achieved clinical pregnancy, experienced a lack of pregnancy or miscarriage and in fertile controls (without steroid treatment). sTNFR1 – soluble TNFR1. Red points indicate measurement before embryo transfer; green, after embryo transfer; dark grey points, fertile control; light grey points, fertile pregnant control. *N* is the numer of women. Black horizontal lines represent medians. P values are calculated by Mann-Whitney test.

There were no significant differences between patients receiving steroid treatment and those that did not in terms of the secretion of soluble TNFR1 (sTNFR1) (**Figure 5A**; **Supplementary Table 9**). The highest amount of soluble TNFR1 was excreted by fertile women who gave birth in the past (median 1843 pg/ml). These women differed in their plasma concentration of sTNFR1 to IVF patients not receiving steroid treatment (p = 0.0069, median 1843 vs. 962.80 pg/ml before ET) and also with patients who did receive steroid treatment (p < 0.0001, median 1843 vs. 676.50 pg/ml before ET; p = 0.0007, median 1843 vs. 998.70 pg/ml after ET).

Among those patients receiving steroid treatment, the lowest levels of sTNFR1 were observed in patients experiencing a lack of pregnancy (median 0.00 and 143.30 pg/ml, before and after ET, respectively; **Figure 5B**; **Supplementary Table 10**). Patients who achieved pregnancy secreted higher levels of sTNFR1 than those with a lack of pregnancy (p <0.0001, median 1134 vs. 0.00 pg/ml before ET; p = 0.0015, median 1207.00 vs. 143.30 pg/ml after ET) the same was observed in the case of patients who experienced miscarriage in comparison to patients with a lack of pregnancy (p = 0.0420, median 414.70 vs. 0.00 pg/ml before ET). When embryo transfer resulted in pregnancy, patients had higher levels of sTNFR1 than those who had miscarried (p = 0.0066, median 1134.00 vs. 414.70 pg/ml before ET and p = 0.0504, median 1207.00 vs. 721.30 pg/ml after ET). All patients differed to the fertile controls in terms of the concentration of sTNFR1, but the most significant distinction was between patients with a lack pregnancy and the fertile controls (p < 0.0001, median 0.00 before ET vs. 1843 pg/ml and p < 0.0001, median 143.30 after ET vs. 1843 pg/ml). These patients also secreted less sTNFR1 than the fertile pregnant controls (p = 0.0005, median 0.00 pg/ml before ET vs. 523.80 pg/ml and p = 0.0162, median 143.30 pg/ml after ET vs. 523.80 pg/ml).

### 3.6 VEGF-A secretion in IVF patients and fertile control groups

**Figure 6:**
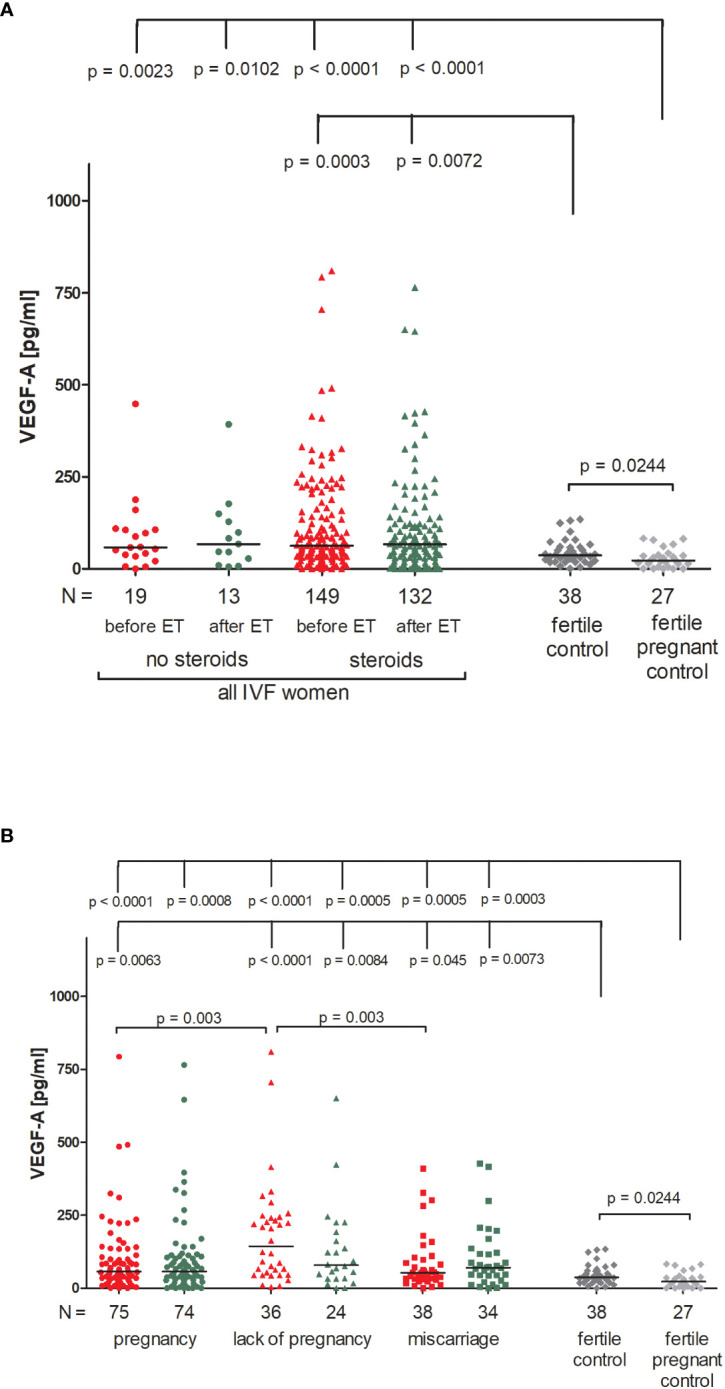
Concentration of plasma VEGF-A measured before and after embryo transfer in all patients and fertile controls. **(A)** Concentration of VEGF-A (pg/ml) measured before and after embryo transfer in patients receiving steroid or no steroid treatment and in fertile controls (without steroid treatment); **(B)** Concentration of VEGF-A (pg/ml) measured before and after embryo transfer in steroid treatment patients who achieved clinical pregnancy, experienced a lack of pregnancy or miscarriage and in fertile controls (without steroid treatment). Red points indicate measurement before embryo transfer; green, after embryo transfer; dark grey points, fertile control; light grey points, fertile pregnant control. *N* is the numer of women. Black horizontal lines represent medians. P values are calculated by Mann-Whitney test.

Patients receiving steroid treatment and patients without steroid treatment had a similar concentration of VEGF-A in blood plasma (**Figure 6A**; **Supplementary Table 11**). The lowest quantities of VEGF-A were detected in the fertile pregnant controls (median 22.62 pg/ml). Significant differences between fertile pregnant control women and patients receiving steroid treatment (p < 0.0001, median 22.62 vs. 62.73 pg/ml before ET and p < 0.0001, median 22.62 vs. 66.30 pg/ml after ET) and patients with no steroid treatment were observed (p = 0.0023, median 22.62 vs. 58.44 pg/ml before ET and p = 0.0102, median 22.62 vs. 67.48 pg/ml after ET). Fertile women who gave birth in the past secreted more VEGF-A than fertile pregnant women (p = 0.0204, median 36.80 vs. 22.62 pg/ml) but less than patients receiving steroid treatment (p = 0.0003, median 36.80 vs. 62.73 pg/ml before ET and p = 0.0072, median 36.80 vs. 66.30 pg/ml after ET).

Patients divided according to their pregnancy outcome had higher levels of VEGF-A than fertile pregnant control women (**Figure 6B**; **Supplementary Table 12**). The most striking difference was found between fertile pregnant women and patients who did not achieve pregnancy (p < 0.0001, median 22.62 vs. 143.10 pg/ml before ET and p = 0.0005, median 22.62 vs. 78.41 pg/ml after ET). We also observed a higher level of VEGF-A in patients who experienced a lack of pregnancy in comparison to patients who became pregnant after IVF-ET (p = 0.003, median 143.10 vs. 57.19 pg/ml before ET) and those who miscarried (p = 0.003, median 143.10 vs. 52.72 pg/ml before ET).

### 3.7 ROC analyses for secretion of soluble TNFR1 and VEGF-A

**Figure 7:**
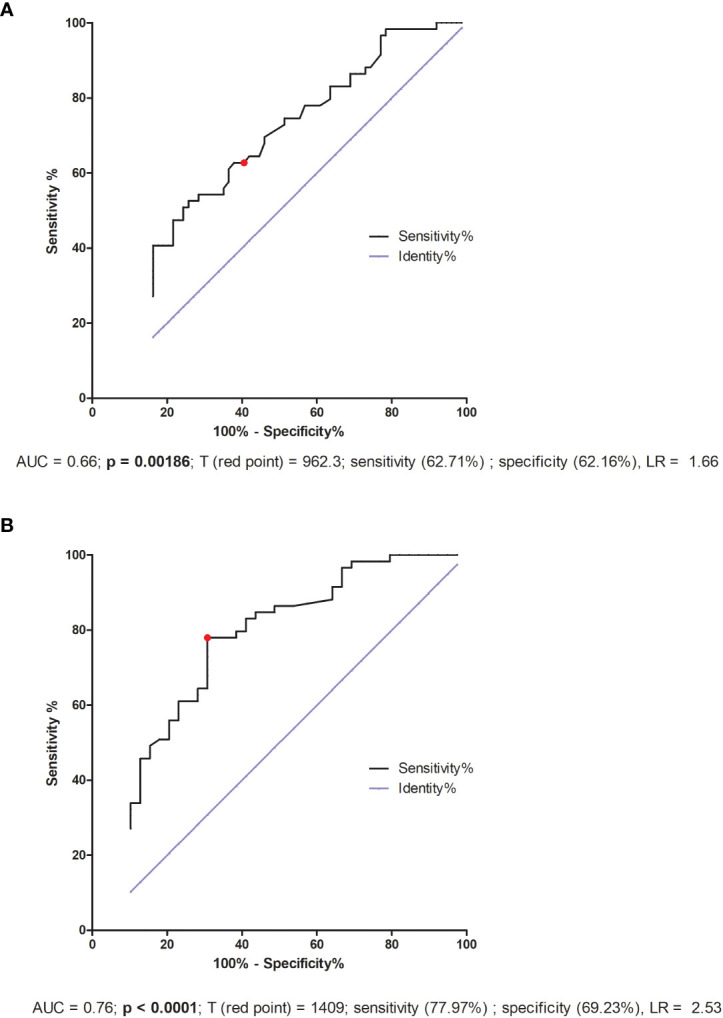
ROC analyses of sTNFR1 concentration in IVF patients and fertile controls. **(A)** Comparison of patients who became pregnant after IVF-ET and maintained it to patients who failed to conceive or had a miscarriage. AUC (Area under curve), T (red point) - limit value, LR – likelihood ratio. **(B)** Comparison of patients who did not become pregnant after IVF-ET to fertile women who gave birth in the past. AUC (Area under curve), T (red point) - limit value, LR – likelihood ratio.

We also performed a receiver–operator curve (ROC) analyses on the secretion of all the examined cytokines, but only in the case of sTNFR1 and VEGF-A did we find statistical significance. We determined the threshold value of sTNFR1 to be 962.3 pg/ml, which allowed us to distinguish IVF patients who achieved and maintained pregnancy from those who did not get pregnant or had a miscarriage (area under the curve (AUC) = 0.66, p = 0.00186, sensitivity 62.71%, specificity 62.16%, and likelihood ratio (LR) = 1.66; **Figure 7A**). When we compared fertile women who gave birth in the past to those who experienced a lack of pregnancy or had miscarriage, the threshold value was 1409 pg/ml (AUC = 0.76, p < 0.0001, sensitivity 77.97%, specificity 69.23%, LR = 2.53; **Figure 7B**).

**Figure 8:**
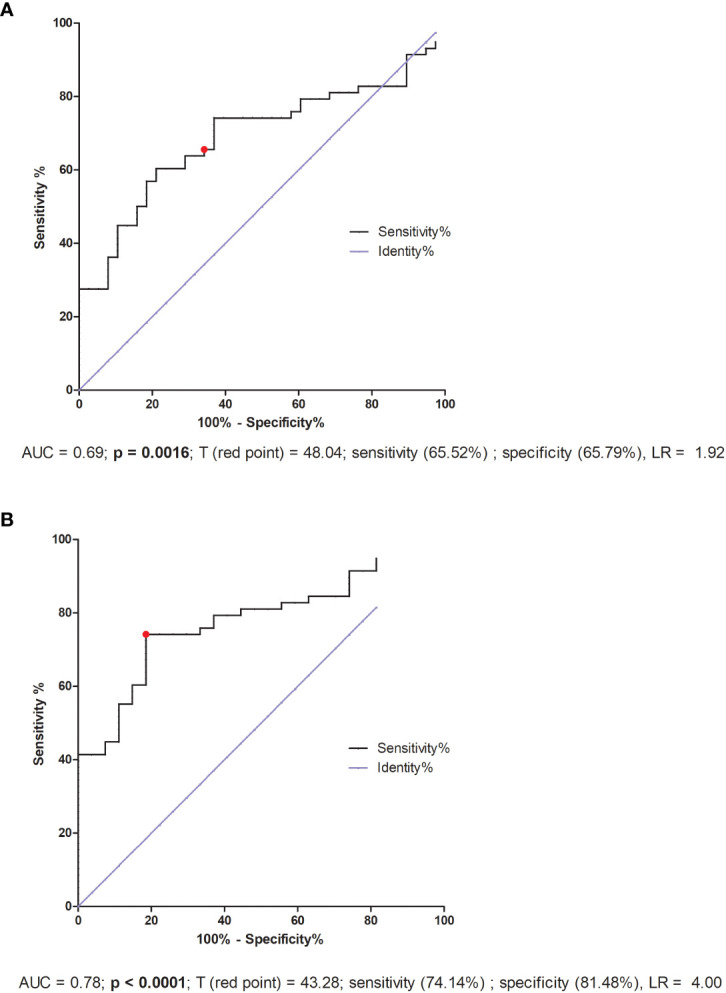
ROC analyses of VEGF-A concentration in IVF patients and fertile controls. **(A)** Comparison of patients who became pregnant after IVF and maintained it to patients who failed to conceive or had a miscarriage. AUC (Area under curve), T (red point) - limit value, LR – likelihood ratio. **(B)** Comparison of patients who did not become pregnant after *in vitro* fertilization to fertile women who gave birth in the past. AUC (Area under curve), T (red point) - limit value, LR – likelihood ratio.

ROC analysis for IVF patients indicated only the lack of significant differences in reproductive success. But the threshold value 48.04 pg/ml was the value differentiating fertile women who gave birth in the past with patients who did not achieve pregnancy or miscarried after IVF-ET (AUC = 0.69, p = 0.0016, sensitivity 65.52%, specificity 65.79%, LR = 1.92; **Figure 8A**). Patients who secreted VEGF-A above this value had a greater risk of miscarriage or failed transfer than women secreting below this value. Additionally, when we compared the fertile pregnant controls with patients who experienced a lack of pregnancy or miscarriage, we achieved a threshold value of 43.28 pg/ml, but the ROC analysis was stronger (AUC = 0.78, p < 0.0001, sensitivity 74.14%, specificity 81.48%, LR = 4.00; **Figure 8B**).

## 4 Discussion

In successful embryo implantation and maintenance of pregnancy, the proper interaction between the embryo and endometrium is needed. Secretion of pro- and anti-inflammatory cytokines and their changing profile is one of the most crucial factors during embryo implantation and throughout the various stages of pregnancy (37, 38). In this study we investigated cytokine and growth factor levels measured in plasma before and after embryo transfer of patients undergoing IVF and also in the fertile controls. Patients being administered the steroid prednisone were compared in terms of the secretion of cytokines with patients who received no treatment with steroid, which may give a new insight into research on recurrent implantation failure and steroid treatment during *in vitro* fertilization. We wanted also to examine whether an individual cytokine level could serve as an indicator of pregnancy outcome after embryo transfer. In addition, comparison with fertile controls identified those factors that may be associated with susceptibility to infertility.

Our study on the plasma level of IFN-ɣ indicates that taking prednisone reduces secretion of this cytokine in patients undergoing IVF-ET. Fertile controls, who did not use this steroid, had a similar level of IFN-ɣ as patients without steroid treatment. It indicates that approximately 0.28 pg/ml of IFN-ɣ must be detectable in women planning pregnancy. The role of IFN-ɣ in pregnancy has been confirmed in other studies (16, 39). IFN-ɣ, which is found in the human endometrium, plays a role in the maintenance of a pregnancy via up-regulating the expression of IL-6, monocyte chemotactic protein (MCP)-1, and macrophage colony stimulating factor (M-CSF) by endometrial stromal cells (40, 41). However, as previously mentioned, during implantation and early pregnancy, there are rapid changes in the immune response in decidua that affect the embryo implantation and pregnancy (42). It is believed that Th1 cytokines like IFN-ɣ and TNF-α are unfavorable to a pregnancy, while Th2 cytokines as IL-4 and IL-10 are significant in the successful development of a fetus. It follows that an increase in the Th1/Th2 ratio may lead to increased cytotoxicity against the embryo and subsequently to implantation failure (43). When a Th1 environment is dominant in the endometrium, uterine NK cells may become killer cells acting upon trophoblast cells and destroying them as a consequence. Thus, proper immune balance is required for embryo implantation (44). Liang et al. reported that both the concentration of IFN-ɣ in blood plasma and also the ratios of pro- and anti-inflammatory cytokines were higher in patients with RIF compared to the control group (45). Most studies have shown that IFN-γ expression is increased in spontaneous abortion or recurrent pregnancy loss patients (46–48). Higher levels of IFN-γ were found in serum samples of women in first trimester of pregnancy who have experienced preeclampsia and fetal growth restriction (49). Therefore, it is considered that inhibiting and regulation of the immune system by immunomodulators or immunosuppressive agents could be an effective treatment strategy to resolve RIF (50, 51). Corticosteroids are often used as immunotherapy for patients suffering from RIF or recurrent miscarriage because of their anti-inflammatory and immune-suppressive properties (52). However, the effect of prednisone on the IFN-γ level did not change in reproductive success of our patients. There were no differences in its secretion in patients achieving pregnancy after ET, patients without pregnancy after ET and also patients experiencing miscarriage.

In the case of IL-10, prednisone treatment increased its concentration in the blood plasma of the patients, while in both the non-treated non-pregnant fertile women and pregnant fertile women, its level was mostly undetectable. Other studies by Dibble et al. and Holmes et al. have reported higher levels of IL-10 in the plasma of pregnant women in comparison to non-pregnant women at different stages of pregnancy and postpartum (53, 54). However, the association between IL-10 concentration in peripheral blood during pregnancy and gestational period is inconclusive. Some studies showed no association (54, 55), while others, for example, by Dibble et al. and Allswede et al. found that plasma IL-10 levels increased with advanced gestational weeks throughout normal pregnancy (53, 56). On the other hand, our pregnant control women were mainly at 25 weeks of gestation and we were not able to detect this cytokine in their plasma. Furthermore, decreased IL-10 levels were associated with pregnancy losses, preeclampsia, and preterm delivery (48, 53, 57, 58). An elegant study was presented by Zhao et al., 2022, who determined the level of anti-inflammatory cytokines and chemokines in the period before and after embryo implantation in patients after IVF (59). During the peri-implantation period (ET+0 to ET+9 days), the serum cytokine profiles were similar between pregnancies which miscarried or did not miscarry. Yet, the profiles from day ET+16 indicated a significant difference concerning an increase of the pro-inflammatory cytokines IL-17, IFN-γ and TNF-α in the miscarriage group, but a sustained increase of anti-inflammatory cytokines IL-10 and TGF-β1 in the live birth group. Moreover, a change in the anti-inflammatory response at 3–6 days after ET occurred in all pregnancies whether they resulted in live birth or miscarriage. Therefore, the researchers concluded that the most exact day for measurement of pro- and anti-inflammatory cytokines monitoring the pregnancy outcome was ET+16. In our study, plasma was collected between 11 to 15 days after embryo transfer. Probably, the differences between our study and the one quoted resulted from the day of material collection for testing, as well as from prednisone treatment. In addition, we did not detect differences in the concentration of all tested cytokines and growth factors between plasma collected before and after embryo transfer. It should also be emphasized that some measurements could not be evaluated, e.g., TNF-α, GM-CSF, IL-4, IL-6, PlGF and VEGF-D. Glucocorticoids acting on antigen presenting cells (APCs) inhibit the production of IL-12, the main inducer of the Th1 response, resulting in a change in the Th1/Th2 balance. IL-12 stimulates IFN-γ production and inhibits IL-4 synthesis by T cells (60). Moreover, glucocorticoid-treated monocytes/macrophages produce significantly less IL-12, leading to a decreased capacity of these cells to induce IFN-γ production by antigen-primed CD4+ T cells (61). In addition, glucocorticoids downregulate the expression of IL-12 receptors on T and NK cells. It has been shown that lymphocyte-derived IL-10 production appears to be upregulated by glucocorticoids. In multiple sclerosis patients with an acute relapse, treatment with glucocorticoids was associated with increased plasma IL-10 secretion (62). A similar steroidal effect was observed in our patients.

Neutrophins like BDNF ensure the survival, differentiation or death of neurons at the embryonic and postnatal stages of development. They also maintain the viability of neurons at a later age (31, 63). Moreover, BDNF and its receptors are expressed in different regions of the human placenta, indicating the diverse functions of BDNF signaling in placental development (64, 65). Our findings point out that fertile pregnant women differ in BDNF secretion in peripheral blood from IVF patients. Moreover, they also differ from fertile women who gave birth in the past. Prednisone administration did not influence the level of BDNF in the plasma of IVF patients. Among our IVF patients, we did not find differences in pregnancy outcome, although the median concentration of BDNF was 8.93 pg/ml in patients experiencing miscarriage and 13.53 pg/ml in patients without a pregnancy. During pregnancy, this level decreased to 0.00 pg/ml for the control group and IVF patients (measured in plasma collected before embryo transfer; **Supplementary Table 6**). A similar observation was found in a study performed by Garces et al. (66). BDNF levels have been reported to be higher in non-pregnant women than in pregnant women. Moreover, they did not vary during menstrual cycle phases and were lowest in pregnant women during the 1^st^ trimester and similar during the 2^nd^ and 3^rd^ trimesters (66). The question arises: Is BDNF taken up from peripheral blood and directed to the uterus when a woman becomes pregnant to fulfill its function in the development of fetus and thus is undetectable in blood of pregnant women? However, a study by Ramer does not support our results (32). Diminished circulating BDNF early in an IVF cycle was associated with implantation and subsequent pregnancy failure (32). The primary difference between our study and their study was the lack of prednisone administration and the time frame for serum collection.

LIF, an IL-6 cytokine family member, is expressed by the endometrium during the menstrual cycle in healthy women, with a remarkable increase during the mid-secretory to late-secretory phase (67–69). It initiates multiple factors and signaling pathways in the luminal epithelium for uterine receptivity and successful implantation (70–72). In our IVF patients, a lack of pregnancy was associated with a decreased level of LIF measured in plasma. Women achieving a pregnancy after IVF secreted LIF at a median concentration over 2 pg/ml. Surprisingly, the median LIF of our pregnant control group was 0.0 pg/ml, and that of women who gave birth in the past was over 2.5 pg/ml. However, we do not have information concerning in which phase of the menstrual cycle were women giving birth in the past. Perhaps LIF levels decline with gestational age. The pregnant women in our study were on average 25 weeks pregnant. Elevated levels of LIF have been observed in the development and progression of tumors (73). Undoubtedly, at the beginning of a pregnancy when trophoblast invasion occurs, elevated levels of LIF are required. Meanwhile, in a study by Basatvat et al., there was no reported difference in LIF gene expression level from the endometrial biopsies of healthy women and IVF-failure patients (74). In another study, a low concentration of LIF in uterine flushing fluid at day 26 was predictive of subsequent implantation and the LIF concentration was significantly lower in the pregnant group (75). Prednisolone treatment reduced the expression of human endometrial stromal fibroblasts (hESFs) cytokines (IL-6, IL-11, IL-18, LIF, and LIFR), however it had no effect on classic decidualization marker gene expression (prolactin or insulin like growth factor binding protein 1) or secretion by decidualized hESF (76).

TNFR1, also known as tumor necrosis factor receptor 1A superfamily member and CD120a, is a ubiquitous membrane receptor that binds tumor necrosis factor alpha (TNF-α). Membrane TNFα, as well as its receptors TNFR1 and TNFR2, after cleavage by the proteinase TACE (TNF-α converting enzyme), occur in a soluble form (sTNFR) in many body fluids. Elevated levels of sTNFR have been observed in serum, plasma and urine and are often found to be in connection with endotoxinemia, infections and malignancies, as well as chronic autoimmune diseases. The shedding of TNF receptors may lead to a transiently reduced cellular response to TNF-α. Depending on the sTNFR concentration, it has been suggested that sTNFRs block the binding of TNF-α to transmembrane TNFR and thus delay biological responses (77). However, it is also possible that shedding of TNFR in inflammation may initiate direct signaling effects through the binding of sTNFR to transmembrane TNF-α (mTNF-α) (78, 79). Therefore, the higher the concentration of circulating sTNFR, the lower the bioavailability of TNF-α, which may reduce the inflammatory response. Thus, it seems reasonable to ask whether it makes sense to administer steroids to patients who have a higher level of sTNFR1? sTNFR1 can fulfill its function by either blocking membrane TNF-α on cells or capturing TNF-α circulating in peripheral blood. The role of sTNFR1 has been described in pre-eclampsia and pregnancy-induced hypertension in several studies (80–85) alongside early miscarriage (86, 87). Our study shows the secretion of sTNFR1 into the plasma tested before embryo transfer and at a very early stage of pregnancy (2-3 weeks after embryo transfer to the uterus) in patients who have undergone an IVF procedure. The lowest sTNFR1 concentration was observed in women who did not become pregnant after ET, and the highest in fertile controls who gave birth in the past. It should be noted that fertile pregnant women secreted sTNFR1 at an intermediate level (median 523.80 pg/ml). These women had an average of 25 weeks of gestation. There is a study showing that sTNFR1 expression decreases with gestational age. In the first trimester, secretion of sTNFR1 and sTNFR2 by placental macrophages were 46- and 260-fold higher than TNF-α concentration, respectively, whereas in the third trimester, a 15- and 1.7-fold excess was observed (88). Our results also indicate that women who secrete at least 1409 pg/ml sTNFR1 have a chance to become pregnant naturally and give birth to a child, while patients after IVF-ET with simultaneous support of the luteal phase and prednisone administration must achieve a concentration of 962.3 pg/ml sTNFR1 in the blood plasma.

VEGF-A (also called VEGF) is a multi-functional factor engaged in the regulation of proliferation, differentiation and survival of endothelial cells and it is also involved in blood vessel permeability. VEGF participates in embryonic vasculogenesis and angiogenesis during pregnancy (27). Progesterone and estrogen significantly promote VEGF production (89, 90). Therefore, in our study IVF patients, who were taking micronized progesterone, secreted more VEGF-A than in our control group. In general, we observed higher VEGF concentrations in IVF patients compared to fertile women, regardless of whether the patients were receiving steroid treatment or not. Moreover, the highest concentrations were observed in women who did not become pregnant after IVF-ET. Similar to our study, Bansal et al. showed that VEGF-A levels were significantly increased in women with RIF compared to healthy controls, however there was no correlation between VEGF-A or VEGF-R1 and the absolute levels of circulating NK cells, CD69 activated NK cells or NK cytotoxicity (91). In contrast to our research, Benkhalifa et al. reported decreased concentration of VEGF in RIF women compared to women with a successful of IVF-ET with an AUC of 0.742 in ROC curve analysis and a cut-off at 55.80 pg/ml (92). The threshold value 43.28 pg/ml of our ROC analysis differentiated fertile pregnant women from IVF patients who did not become pregnant or who miscarried. Our study differs from those previously mentioned not only in the fact that patients took prednisone and progesterone alongside differences in group size but also in that they did not compare the IVF patients with a healthy fertile control group. Thus, the analysis of VEGF levels should take into consideration the impact of estrogen, progesterone and other potential influential factors to understand the role of VEGF during embryo implantation.

One of the beneficial effects of prednisolone might be its suppressive impact on uterine NK cells. A study by Cooper et al. showed a relatively high prevalence of increased uNK cells in women with recurrent reproductive failure and confirmed the role of prednisolone in reducing uNK cell concentrations (93). Nevertheless, they did not find any evidence for a significant beneficial effect of prednisolone therapy on pregnancy outcomes. In turn, Giulini et al. suggest the advantage of prednisone and antibiotic treatment on the live birth rate in a subsequent IVF cycle (94). Indeed, the reported effectiveness of prednisolone/prednisone in prevention of miscarriage/recurrent implantation failure is highly variable between studies (93, 95–100). Therefore, the Practice Committee of the American Society for Reproductive Medicine since 2018 has not recommended the routine use of corticosteroids during ovarian stimulation to improve the outcome of live birth in ART cycles in the general population (101). ASRM has also not recommended the routine use of corticosteroids during the implantation window to improve the outcome of live births in ART cycles in the general population. In addition, the latest recommendations of the European Society of Human Reproduction and Embryology concerning recurrent implantation failure treatment do not contain guidelines for steroid treatment (102). According to the latest European recommendations (strong quality evidence), corticosteroids should be administered to women at a gestational age between 24 + 0 and 33 + 6 weeks, when preterm birth is expected in the next seven days. This treatment reduced neonatal mortality and morbidity by approximately 20% and neonatal respiratory distress syndrome by approximately 30% (103).

However, our research has limitations. Firstly, we tested only selected immunological factors that did not give a full picture of the immunological condition of the women studied. One of the weaknesses of our study was also the lack of information about the day of the menstrual cycle of the tested women who gave birth in the past, which may have influenced the results obtained. However, these were women of reproductive age (except for 1). Therefore, we added a control who was naturally pregnant at the time of blood sampling to compare the women in terms of conception method. Moreover, the group of women not treated with the steroid (N = 19 before ET) seems small compared to that treated. Despite the use of modern Luminex technology, we were unable to obtain unequivocal conclusions for all analytes tested. Panels of tested analytes need to be more refined.

## Conclusions

- Fertile women present a different profile of pro- and anti-inflammatory cytokines and growth factors from patients with RIF.
- Women who secrete at least 1409 pg/ml sTNFR1 have a chance to become pregnant naturally and give birth to a child, while patients after IVF-ET with simultaneous luteal phase support and receiving steroid treatment must achieve a concentration of 962.3 pg/ml sTNFR1 in the blood plasma.
- IVF patients secreting VEGF-A above 43.28 pg/ml have a greater risk of miscarriage or failed transfer than women secreting below this value.
- The use of steroid treatment by infertile patients should be carefully considered due to the changes they cause in the immune environment of the uterus and in the peripheral blood.

## Data availability statement

The original contributions presented in the study are included in the article/**Supplementary Materials**, further inquiries can be directed to the corresponding author.

## Ethics statement

The studies involving humans were approved by Ethics Committee of the Polish Mothers’ Memorial Hospital–Research Institute in Łódź (No: 25/2019). The studies were conducted in accordance with the local legislation and institutional requirements. The participants provided their written informed consent to participate in this study.

## Author contributions

KP, IN conceived and designed the experiments. KP, MD performed the experiments. KP, MD, IN, PR analyzed the data. PR, MR contributed to patient and control recruitment. KP and IN wrote the paper. KB-K contributed to the research methodology, provided equipment for the study and critically reviewed the manuscript. All authors contributed to the article and approved the submitted version.
