# Supplementary material for: Pro- and anti-inflammatory cytokines and growth factors in patients undergoing in vitro fertilization procedure treated with prednisone
Source: Front Immunol. 2023 Sep 6;14:1250488. doi: 10.3389/fimmu.2023.1250488 (PMC10511889; doi:10.3389/fimmu.2023.1250488)
Supplement: Supplementary file 1 [file Table_1.docx]

**Supplementary Table 1** IFN-ɣ value (pg/ml) measured before and after IVF embryo transfer, both in those patients who received steroid treatment and those who did not, as well as in the fertile controls.

ET – embryo transfer; p values are calculated by Mann-Whitney test:

**Without steroid treatment patients vs steroid treatment patients before ET:** ^a^ p = 0.0005;

**Without steroid treatment patients vs steroid treatment patients after ET:** ^b^ p < 0.0001;

**Without steroid treatment patients after ET vs fertile control:** ^c^ p = 0.0041;

**Without steroid treatment patients after ET vs fertile pregnant control:** ^d^ p = 0.0148;

**Steroid treatment patients before ET vs fertile control:** ^e^ p < 0.0001;

**Steroid treatment patients before ET vs fertile pregnant control: ^f^** p < 0.0001;

**Steroid treatment patients after ET vs fertile control:** ^g^ p < 0.0001;

**Steroid treatment after ET vs fertile pregnant control:** ^h^ p < 0.0001.

| **Study group** | **IVF patients** | | | | **Fertile control** | **Fertile pregnant control** |
| --- | --- | --- | --- | --- | --- | --- |
| **Treatment** | **Without steroid** | | **Steroid** | |  |  |
| **Before or after IVF-ET** | **before** | **after** | **before** | **after** |  |  |
| Number of women | 10 | 10 | 113 | 106 | 38 | 27 |
| Minimum | 0.00 | 0.00 | 0.00 | 0.00 | 0.00 | 0.26 |
| 25% Percentile | 0.20 | 0.13 | 0.00 | 0.00 | 0.28 | 0.27 |
| Median | **0.28^a^** | **0.26^b, c, d^** | **0.00^e, f^** | **0.00^g, h^** | 0.29 | 0.28 |
| 75% Percentile | 0.37 | 0.28 | 0.00 | 0.00 | 0.31 | 0.31 |
| Maximum | 0.98 | 0.70 | 1.09 | 1.62 | 0.53 | 0.62 |
| Mean | 0.32 | 0.25 | 0.12 | 0.11 | 0.28 | 0.30 |
| Std. Deviation | 0.28 | 0.19 | 0.26 | 0.28 | 0.08 | 0.07 |
| Std. Error | 0.09 | 0.06 | 0.02 | 0.03 | 0.01 | 0.01 |
| Lower 95% CI of mean | 0.12 | 0.11 | 0.07 | 0.06 | 0.26 | 0.28 |
| Upper 95% CI of mean | 0.52 | 0.39 | 0.17 | 0.16 | 0.31 | 0.33 |
| D'Agostino & Pearson omnibus normality test K^2^ | 8.09 | 6.97 | 52.77 | 82.82 | 30.96 | 48.60 |
