# Supplementary material for: Pro- and anti-inflammatory cytokines and growth factors in patients undergoing in vitro fertilization procedure treated with prednisone
Source: Front Immunol. 2023 Sep 6;14:1250488. doi: 10.3389/fimmu.2023.1250488 (PMC10511889; doi:10.3389/fimmu.2023.1250488)
Supplement: Supplementary file 2 [file Table_2.docx]

**Supplementary Table 2** IFN-ɣ value (pg/ml) measured before and after IVF embryo transfer in all patients who received steroid treatment, including those who achieved pregnancy, experienced a lack of pregnancy or miscarriage, as well as in the fertile controls.

ET – embryo transfer; p values are calculated by Mann-Whitney test:

**Pregnancy before ET vs fertile control:** ^a^ p < 0.0001;

**Pregnancy before ET vs fertile pregnant control:** ^b^ p < 0.0001;

**Pregnancy after ET vs fertile control:** ^c^ p < 0.0001;

**Pregnancy after ET vs fertile pregnant control:** ^d^ p < 0.0001;

**Lack of pregnancy before ET vs fertile control:** ^e^ p = 0.0002;

**Lack of pregnancy before ET vs fertile pregnant control:** ^f^ p = 0.0002;

**Lack of pregnancy after ET vs fertile control:** ^g^ p = 0.0001;

**Lack of pregnancy after ET vs fertile pregnant control:** ^h^ p < 0.0001;

**Miscarriage before ET vs fertile control:** ^i^ p < 0.0001;

**Miscarriage before ET vs fertile pregnant control: ^j^** p < 0.0001;

**Miscarriage after ET vs fertile control:** ^k^ p < 0.0001;

**Miscarriage after ET vs fertile pregnant control: ^l^** p < 0.0001.

| **Study group** | **IVF steroid treatment patients** | | | | | | **Fertile control** | **Fertile pregnant control** |
| --- | --- | --- | --- | --- | --- | --- | --- | --- |
| **Pregnancy outcome** | **Pregnancy** | | **Lack of pregnancy** | | **Miscarriage** | |  |  |
| **Before or after IVF-ET** | **before** | **after** | **before** | **after** | **before** | **after** |  |  |
| Number of women | 68 | 65 | 15 | 15 | 30 | 26 | 38 | 27 |
| Minimum | 0.00 | 0.00 | 0.00 | 0.00 | 0.00 | 0.00 | 0.00 | 0.26 |
| 25% Percentile | 0.00 | 0.00 | 0.00 | 0.00 | 0.00 | 0.00 | 0.28 | 0.27 |
| Median | **0.00^a, b^** | **0.00^c, d^** | **0.00^e, f^** | **0.00^g, h^** | **0.00^i, j^** | **0.00^k, l^** | 0.29 | 0.28 |
| 75% Percentile | 0.00 | 0.00 | 0.00 | 0.00 | 0.19 | 0.00 | 0.31 | 0.31 |
| Maximum | 1.09 | 1.62 | 0.78 | 0.93 | 0.90 | 0.87 | 0.53 | 0.62 |
| Mean | 0.11 | 0.12 | 0.11 | 0.12 | 0.16 | 0.08 | 0.28 | 0.30 |
| Std. Deviation | 0.25 | 0.29 | 0.26 | 0.30 | 0.30 | 0.23 | 0.08 | 0.07 |
| Std. Error | 0.03 | 0.04 | 0.07 | 0.08 | 0.05 | 0.05 | 0.01 | 0.01 |
| Lower 95% CI of mean | 0.05 | 0.05 | -0.03 | -0.04 | 0.05 | -0.01 | 0.26 | 0.28 |
| Upper 95% CI of mean | 0.17 | 0.19 | 0.26 | 0.29 | 0.27 | 0.17 | 0.31 | 0.33 |
| D'Agostino & Pearson omnibus normality test K^2^ | 45.92 | 62.29 | 15.64 | 18.69 | 12.02 | 30.49 | 30.96 | 48.60 |
