# Supplementary material for: Pro- and anti-inflammatory cytokines and growth factors in patients undergoing in vitro fertilization procedure treated with prednisone
Source: Front Immunol. 2023 Sep 6;14:1250488. doi: 10.3389/fimmu.2023.1250488 (PMC10511889; doi:10.3389/fimmu.2023.1250488)
Supplement: Supplementary file 3 [file Table_3.docx]

**Supplementary Table 3** IL-10 value (pg/ml) measured before and after IVF embryo transfer, both in those patients who received steroid treatment and those who did not, as well as in the fertile controls.

ET – embryo transfer; p values are calculated by Mann-Whitney test:

**Without steroid treatment patients vs steroid treatment patients before ET:** ^a^ p = 0.0063;

**Without steroid treatment patients before ET vs fertile control:** ^b^ p = 0.0027;

**Without steroid treatment patients vs steroid treatment patients after ET:** ^c^ p = 0.0007;

**Without steroid treatment patients after ET vs fertile control:** ^d^ p = 0.009;

**Receiving steroid patients before ET vs fertile control:** ^e^ p < 0.0001;

**Receiving steroid patients after ET vs fertile control: ^f^** p < 0.0001.

| **Study group** | **IVF patients** | | | | **Fertile control** | **Fertile pregnant control** |
| --- | --- | --- | --- | --- | --- | --- |
| **Treatment** | **Without steroid** | | **Steroid** | |  |  |
| **Before or after IVF-ET** | **before** | **after** | **before** | **after** |  |  |
| Number of women | 9 | 8 | 115 | 106 | 40 | 27 |
| Minimum | 0.00 | 0.00 | 0.00 | 0.00 | 0.00 | 0.00 |
| 25% Percentile | 0.00 | 0.00 | 0.07 | 0.19 | 0.00 | 0.00 |
| Median | **0.05^a, b^** | **0.04^c, d^** | **0.31^e^** | **0.34^f^** | 0.00 | 0.00 |
| 75% Percentile | 0.11 | 0.10 | 0.54 | 0.78 | 0.00 | 0.00 |
| Maximum | 0.38 | 0.17 | 2.39 | 5.85 | 0.87 | 0.00 |
| Mean | 0.08 | 0.05 | 0.37 | 0.63 | 0.06 | 0.00 |
| Std. Deviation | 0.12 | 0.06 | 0.39 | 0.87 | 0.17 | 0.00 |
| Std. Error | 0.04 | 0.02 | 0.04 | 0.08 | 0.03 | 0.00 |
| Lower 95% CI of mean | -0.01 | 0.00 | 0.30 | 0.46 | 0.00 | 0.00 |
| Upper 95% CI of mean | 0.17 | 0.11 | 0.45 | 0.80 | 0.11 | 0.00 |
| D'Agostino & Pearson omnibus normality test K^2^ | 16.48 | 1.14 | 78.36 | 98.94 | 55.23 | - |
