# Supplementary material for: Pro- and anti-inflammatory cytokines and growth factors in patients undergoing in vitro fertilization procedure treated with prednisone
Source: Front Immunol. 2023 Sep 6;14:1250488. doi: 10.3389/fimmu.2023.1250488 (PMC10511889; doi:10.3389/fimmu.2023.1250488)
Supplement: Supplementary file 4 [file Table_4.docx]

**Supplementary Table 4** IL-10 values (in pg/ml) measured before and after IVF embryo transfer in all patients who received steroid treatment, including those who achieved pregnancy, experienced a lack of pregnancy or miscarriage, as well as in the fertile controls.

ET – embryo transfer; p values are calculated by Mann-Whitney test:

**Pregnancy before ET vs fertile control:** ^a^ p < 0.0001;

**Pregnancy after ET vs fertile control:** ^b^ p < 0.0001;

**Lack of pregnancy before ET vs fertile control:** ^c^ p < 0.0001;

**Lack of pregnancy after ET vs fertile control:** ^d^ p < 0.0001;

**Miscarriage before ET vs fertile control:** ^e^ p < 0.0001;

**Miscarriage after ET vs fertile control:** ^f^ p < 0.0001.

| **Study group** | **IVF steroid treatment patients** | | | | | | **Fertile control** | **Fertile pregnant control** |
| --- | --- | --- | --- | --- | --- | --- | --- | --- |
| **Pregnancy outcome** | **Pregnancy** | | **Lack of pregnancy** | | **Miscarriage** | |  |  |
| **Before or after IVF-ET** | **before** | **after** | **before** | **after** | **before** | **after** |  |  |
| Number of women | 71 | 66 | 15 | 12 | 29 | 28 | 40 | 27 |
| Minimum | 0.00 | 0.00 | 0.00 | 0.00 | 0.00 | 0.00 | 0.00 | 0.00 |
| 25% Percentile | 0.08 | 0.23 | 0.00 | 0.19 | 0.16 | 0.09 | 0.00 | 0.00 |
| Median | **0.34^a^** | **0.35^b^** | **0.24^c^** | **0.50^d^** | **0.30^e^** | **0.24^f^** | 0.00 | 0.00 |
| 75% Percentile | 0.54 | 0.83 | 0.54 | 1.01 | 0.42 | 0.63 | 0.00 | 0.00 |
| Maximum | 1.28 | 5.85 | 2.39 | 1.52 | 2.21 | 3.19 | 0.87 | 0.00 |
| Mean | 0.37 | 0.68 | 0.42 | 0.61 | 0.37 | 0.53 | 0.06 | 0.00 |
| Std. Deviation | 0.31 | 0.96 | 0.63 | 0.50 | 0.42 | 0.78 | 0.17 | 0.00 |
| Std. Error | 0.04 | 0.12 | 0.16 | 0.14 | 0.08 | 0.15 | 0.03 | 0.00 |
| Lower 95% CI of mean | 0.29 | 0.44 | 0.08 | 0.29 | 0.21 | 0.23 | 0.00 | 0.00 |
| Upper 95% CI of mean | 0.44 | 0.91 | 0.77 | 0.92 | 0.53 | 0.84 | 0.11 | 0.00 |
| D'Agostino & Pearson omnibus normality test K^2^ | 7.64 | 72.37 | 22.76 | 1.326 | 44.43 | 29.67 | 55.23 | - |
