# Supplementary material for: Pro- and anti-inflammatory cytokines and growth factors in patients undergoing in vitro fertilization procedure treated with prednisone
Source: Front Immunol. 2023 Sep 6;14:1250488. doi: 10.3389/fimmu.2023.1250488 (PMC10511889; doi:10.3389/fimmu.2023.1250488)
Supplement: Supplementary file 5 [file Table_5.docx]

**Supplementary Table 5** BDNF value (pg/ml) measured before and after IVF embryo transfer in all patients, both those who received steroid treatment and those who did not, as well as in the fertile controls.

ET – embryo transfer; p values are calculated by Mann-Whitney test:

**Without steroid treatment patients before ET vs fertile pregnant control:** ^a^ p = 0.0047;

**Steroid treatment patients before ET vs fertile pregnant control:** ^b^ p = 0.0062;

**Steroid treatment patients after ET vs fertile pregnant control:** ^c^ p = 0.0006;

**Fertile control vs fertile pregnant control:** ^d^ p = 0.0106.

| **Study group** | **IVF patients** | | | | **Fertile control** | **Fertile pregnant control** |
| --- | --- | --- | --- | --- | --- | --- |
| **Treatment** | **Without steroid** | | **steroid** | |  |  |
| **Before or after IVF-ET** | **before** | **after** | **before** | **after** |  |  |
| Number of women | 18 | 11 | 148 | 131 | 40 | 27 |
| Minimum | 0.00 | 0.00 | 0.00 | 0.00 | 0.00 | 0.00 |
| 25% Percentile | 0.00 | 0.00 | 0.00 | 0.00 | 0.00 | 0.00 |
| Median | **6.52^a^** | 0.00 | **0.00^b^** | **5.38^c^** | **0.30^d^** | 0.00 |
| 75% Percentile | 25.38 | 26.72 | 29.32 | 38.75 | 18.42 | 0.00 |
| Maximum | 84.89 | 103.40 | 227.70 | 250.10 | 169.10 | 42.54 |
| Mean | 17.84 | 17.87 | 21.87 | 26.86 | 19.41 | 3.58 |
| Std. Deviation | 25.34 | 35.08 | 40.45 | 46.26 | 39.76 | 9.79 |
| Std. Error | 5.97 | 10.58 | 3.33 | 4.04 | 6.29 | 1.88 |
| Lower 95% CI of mean | 5.24 | -5.70 | 15.30 | 18.87 | 6.70 | -0.30 |
| Upper 95% CI of mean | 30.44 | 41.43 | 28.44 | 34.86 | 32.13 | 7.45 |
| D'Agostino & Pearson omnibus normality test K^2^ | 9.97 | 11.55 | 110.50 | 90.18 | 36.65 | 38.77 |
