# Supplementary material for: Pro- and anti-inflammatory cytokines and growth factors in patients undergoing in vitro fertilization procedure treated with prednisone
Source: Front Immunol. 2023 Sep 6;14:1250488. doi: 10.3389/fimmu.2023.1250488 (PMC10511889; doi:10.3389/fimmu.2023.1250488)
Supplement: Supplementary file 6 [file Table_6.docx]

**Supplementary Table 6** BDNF values (pg/ml) measured before and after IVF embryo transfer in all patients receiving steroid treatment, including those who achieved pregnancy, experienced a lack of pregnancy or miscarriage, as well as in the fertile controls.

ET – embryo transfer; p values are calculated by Mann-Whitney test:

**Pregnancy before ET vs fertile pregnant control:** ^a^ p = 0.0358;

**Pregnancy after ET vs fertile pregnant control:** ^b^ p = 0.003;

**Lack of pregnancy before ET vs fertile pregnant control:** ^c^ p = 0.0047;

**Lack of pregnancy after ET vs fertile pregnant control:** ^d^ p = 0.0011;

**Miscarriage before ET vs fertile pregnant control:** ^e^ p = 0.0048;

**Miscarriage after ET vs fertile pregnant control:** ^f^ p = 0.0019;

**Fertile control vs fertile pregnant control:** ^g^ p = 0.0106.

| **Study group** | **IVF steroid treatment patients** | | | | | | **Fertile control** | **Fertile pregnant control** |
| --- | --- | --- | --- | --- | --- | --- | --- | --- |
| **Pregnancy outcome** | **Pregnancy** | | **Lack of pregnancy** | | **Miscarriage** | |  |  |
| **Before or after IVF-ET** | **before** | **after** | **before** | **after** | **before** | **after** |  |  |
| Number of women | 75 | 74 | 35 | 25 | 38 | 32 | 40 | 27 |
| Minimum | 0.00 | 0.00 | 0.00 | 0.00 | 0.00 | 0.00 | 0.00 | 0.00 |
| 25% Percentile | 0.00 | 0.00 | 0.00 | 0.00 | 0.00 | 0.00 | 0.00 | 0.00 |
| Median | **0.00^a^** | **1.63^b^** | **0.11^c^** | **13.53^d^** | **0.94^e^** | **8.93^f^** | **0.30^g^** | 0.00 |
| 75% Percentile | 24.74 | 25.60 | 28.99 | 46.32 | 35.84 | 52.18 | 18.42 | 0.00 |
| Maximum | 164.10 | 121.90 | 209.60 | 128.10 | 227.70 | 250.10 | 169.10 | 42.54 |
| Mean | 18.74 | 17.75 | 29.34 | 27.60 | 21.17 | 47.35 | 19.41 | 3.58 |
| Std. Deviation | 33.73 | 27.50 | 53.19 | 38.43 | 39.40 | 73.60 | 39.76 | 9.79 |
| Std. Error | 3.90 | 3.20 | 8.99 | 7.69 | 6.39 | 13.01 | 6.29 | 1.88 |
| Lower 95% CI of mean | 10.98 | 11.38 | 11.07 | 11.74 | 8.22 | 20.81 | 6.70 | -0.30 |
| Upper 95% CI of mean | 26.51 | 24.12 | 47.61 | 43.47 | 34.12 | 73.88 | 32.13 | 7.45 |
| D'Agostino & Pearson omnibus normality test K^2^ | 44.98 | 31.90 | 30.72 | 12.06 | 64.41 | 15.01 | 36.65 | 38.77 |
