# Supplementary material for: Pro- and anti-inflammatory cytokines and growth factors in patients undergoing in vitro fertilization procedure treated with prednisone
Source: Front Immunol. 2023 Sep 6;14:1250488. doi: 10.3389/fimmu.2023.1250488 (PMC10511889; doi:10.3389/fimmu.2023.1250488)
Supplement: Supplementary file 7 [file Table_7.docx]

**Supplementary Table 7** LIF values (pg/ml) measured before and after IVF embryo transfer in all patients, both those who received steroid treatment and those who did not, as well as in the fertile controls.

ET – embryo transfer; p values are calculated by Mann-Whitney test:

**Without steroid treatment patients before ET vs fertile pregnant control:** ^a^ p = 0.0221;

**Without steroid treatment patients after ET vs fertile control:** ^b^ p = 0.0320;

**Without steroid treatment patients after ET vs fertile pregnant control:** ^c^ p = 0.0014;

**Steroid treatment patients before ET vs fertile pregnant control:** ^d^ p = 0.0365;

**Steroid treatment patients after ET vs fertile pregnant control:** ^e^ p = 0.0033;

**Fertile control vs fertile pregnant control:** ^f^ p = 0.0023.

| **Study group** | **IVF patients** | | | | **Fertile control** | **Fertile pregnant control** |
| --- | --- | --- | --- | --- | --- | --- |
| **Treatment** | **Without steroid** | | **Steroid** | |  |  |
| **Before or after IVF-ET** | **before** | **after** | **before** | **after** |  |  |
| Number of women | 19 | 12 | 148 | 133 | 40 | 27 |
| Minimum | 0.00 | 0.00 | 0.00 | 0.00 | 0.00 | 0.00 |
| 25% Percentile | 0.00 | 2.25 | 0.00 | 0.00 | 0.99 | 0.00 |
| Median | **4.62^a^** | **4.96^b, c^** | **1.38^d^** | **2.00^e^** | **2.55^f^** | 0.00 |
| 75% Percentile | 5.48 | 8.52 | 4.79 | 5.14 | 3.40 | 2.55 |
| Maximum | 10.00 | 13.77 | 14.47 | 22.71 | 19.37 | 10.88 |
| Mean | 3.43 | 5.62 | 2.55 | 3.35 | 3.43 | 1.59 |
| Std. Deviation | 3.16 | 4.19 | 3.22 | 4.06 | 4.16 | 2.89 |
| Std. Error | 0.73 | 1.21 | 0.26 | 0.35 | 0.66 | 0.56 |
| Lower 95% CI of mean | 1.90 | 2.96 | 2.02 | 2.65 | 2.10 | 0.44 |
| Upper 95% CI of mean | 4.95 | 8.28 | 3.07 | 4.04 | 4.76 | 2.73 |
| D'Agostino & Pearson omnibus normality test K^2^ | 1.63 | 0.55 | 44.88 | 64.17 | 35.32 | 22.50 |
