# Supplementary material for: Pro- and anti-inflammatory cytokines and growth factors in patients undergoing in vitro fertilization procedure treated with prednisone
Source: Front Immunol. 2023 Sep 6;14:1250488. doi: 10.3389/fimmu.2023.1250488 (PMC10511889; doi:10.3389/fimmu.2023.1250488)
Supplement: Supplementary file 8 [file Table_8.docx]

**Supplementary Table 8** LIF values (pg/ml) measured before and after IVF embryo transfer in all patients who receiving steroid treatment, including those who achieved pregnancy, experienced a lack of pregnancy or miscarriage, as well as in the fertile controls.

ET – embryo transfer; p values are calculated by Mann-Whitney test:

**Pregnancy before ET vs fertile pregnant control:** ^a^ p = 0.0206;

**Pregnancy after ET vs fertile pregnant control:** ^b^ p = 0.0031;

**Pregnancy after ET vs lack of pregnancy after ET:** ^c^ p = 0.0063;

**Lack of pregnancy before ET vs fertile control:** ^d^ p = 0.0024;

**Lack of pregnancy after ET vs fertile control:** ^e^ p = 0.0037;

**Lack of pregnancy after ET vs miscarriage after ET:** ^f^ p = 0.0058;

**Miscarriage after ET vs fertile pregnant control:** ^g^ p = 0.001;

**Fertile control vs fertile pregnant control:** ^h^ p = 0.0023.

| **Study group** | **IVF steroid treatment patients** | | | | | | **Fertile control** | **Fertile pregnant control** |
| --- | --- | --- | --- | --- | --- | --- | --- | --- |
| **Pregnancy outcome** | **Pregnancy** | | **Lack of pregnancy** | | **Miscarriage** | |  |  |
| **Before or after IVF-ET** | **before** | **after** | **before** | **after** | **before** | **after** |  |  |
| Number of women | 74 | 73 | 36 | 25 | 38 | 34 | 40 | 27 |
| Minimum | 0.00 | 0.00 | 0.00 | 0.00 | 0.00 | 0.00 | 0.00 | 0.00 |
| 25% Percentile | 0.00 | 0.00 | 0.00 | 0.00 | 0.00 | 0.45 | 0.99 | 0.00 |
| Median | **1.73^a^** | **2.27^b, c^** | **0.40^d^** | **0.00^e, f^** | 0.85 | **3.27^g^** | **2.55^h^** | 0.00 |
| 75% Percentile | 5.07 | 5.26 | 2.29 | 1.99 | 5.00 | 5.48 | 3.40 | 2.55 |
| Maximum | 14.47 | 14.43 | 11.30 | 12.61 | 13.12 | 17.56 | 19.37 | 10.88 |
| Mean | 2.90 | 3.42 | 1.76 | 1.62 | 2.61 | 3.90 | 3.43 | 1.59 |
| Std. Deviation | 3.41 | 3.62 | 2.89 | 2.83 | 3.11 | 4.18 | 4.16 | 2.89 |
| Std. Error | 0.40 | 0.42 | 0.48 | 0.57 | 0.50 | 0.72 | 0.66 | 0.56 |
| Lower 95% CI of mean | 2.11 | 2.57 | 0.78 | 0.45 | 1.59 | 2.44 | 2.10 | 0.44 |
| Upper 95% CI of mean | 3.69 | 4.26 | 2.73 | 2.79 | 3.63 | 5.36 | 4.76 | 2.73 |
| D'Agostino & Pearson omnibus normality test K^2^ | 23.93 | 18.29 | 26.88 | 34.05 | 12.01 | 19.56 | 35.32 | 22.50 |
