# Supplementary material for: Pro- and anti-inflammatory cytokines and growth factors in patients undergoing in vitro fertilization procedure treated with prednisone
Source: Front Immunol. 2023 Sep 6;14:1250488. doi: 10.3389/fimmu.2023.1250488 (PMC10511889; doi:10.3389/fimmu.2023.1250488)
Supplement: Supplementary file 9 [file Table_9.docx]

**Supplementary Table 9** Soluble TNFR1 value (pg/ml) measured in all patients before and after IVF embryo transfer, both in those who received steroid treatment and those who did not, as well as in the fertile controls.

ET – embryo transfer; p values are calculated by Mann-Whitney test:

**Without steroid treatment patients before ET vs fertile control:** ^a^ p = 0.0069;

**Steroid treatment patients before ET vs fertile control:** ^b^ p < 0.0001;

**Steroid treatment patients after ET vs fertile control:** ^c^ p = 0.0007.

| **Study group** | **IVF patients** | | | | **Fertile control** | **Fertile pregnant control** |
| --- | --- | --- | --- | --- | --- | --- |
| **Treatment** | **Without steroid** | | **Steroid** | |  |  |
| **Before or after IVF-ET** | **before** | **after** | **before** | **after** |  |  |
| Number of women | 19 | 13 | 149 | 133 | 39 | 27 |
| Minimum | 0.00 | 0.00 | 0.00 | 0.00 | 0.00 | 0.00 |
| 25% Percentile | 425.50 | 1033.00 | 0.00 | 140.80 | 910.60 | 311.70 |
| Median | **962.80^a^** | 1177.00 | **676.50^b^** | **998.70^c^** | 1843.00 | 523.80 |
| 75% Percentile | 1389.00 | 1536.00 | 1685.00 | 1687.00 | 2444.00 | 2100.00 |
| Maximum | 2279.00 | 2785.00 | 3948.00 | 4123.00 | 4956.00 | 4575.00 |
| Mean | 962.50 | 1291.00 | 926.80 | 1066.00 | 1850.00 | 1280.00 |
| Std. Deviation | 620.70 | 645.00 | 972.40 | 985.60 | 1320.00 | 1419.00 |
| Std. Error | 142.40 | 178.90 | 79.67 | 85.46 | 211.40 | 273.10 |
| Lower 95% CI of mean | 663.30 | 901.60 | 769.40 | 896.80 | 1422.00 | 718.50 |
| Upper 95% CI of mean | 1262.00 | 1681.00 | 1084.00 | 1235.00 | 2278.00 | 1841.00 |
| D'Agostino & Pearson omnibus normality test K^2^ | 0.45 | 4.10 | 16.34 | 11.97 | 2.59 | 6.59 |
