# Supplementary material for: Pro- and anti-inflammatory cytokines and growth factors in patients undergoing in vitro fertilization procedure treated with prednisone
Source: Front Immunol. 2023 Sep 6;14:1250488. doi: 10.3389/fimmu.2023.1250488 (PMC10511889; doi:10.3389/fimmu.2023.1250488)
Supplement: Supplementary file 10 [file Table_10.docx]

**Supplementary Table 10** Soluble TNFR1 value (pg/ml) measured before and after IVF embryo transfer in all patients who received steroid treatment, including those who achieved pregnancy, experienced a lack of pregnancy or miscarriage, as well as in the fertile controls.

ET – embryo transfer; p values are calculated by Mann-Whitney test:

**Pregnancy before ET vs lack of pregnancy before ET:** ^a^ p < 0.0001;

**Pregnancy before ET vs miscarriage before ET:** ^b^ p = 0.0066;

**Pregnancy before ET vs fertile control:** ^c^ p = 0.0341;

**Pregnancy after ET vs lack of pregnancy after ET:** ^d^ p = 0.0015;

**Pregnancy after ET vs miscarriage after ET:** ^e^ p = 0.0504;

**Pregnancy after ET vs fertile control:** ^f^ p = 0.0395;

**Lack of pregnancy before ET vs miscarriage before:** ^g^ p = 0.0420;

**Lack of pregnancy before ET vs fertile control:** ^h^ p < 0.0001;

**Lack of pregnancy before ET vs fertile pregnant control:** ^i^ p = 0.0005;

**Lack of pregnancy after ET vs fertile control:** ^j^ p < 0.0001;

**Lack of pregnancy after ET vs fertile pregnant control:** ^k^ p = 0.0162;

**Miscarriage before ET vs fertile control:** ^l^ p < 0.0001;

**Miscarriage after ET vs fertile control:** ^m^ p = 0.001.

| **Study group** | **IVF steroid treatment patients** | | | | | | **Fertile control** | **Fertile pregnant control** |
| --- | --- | --- | --- | --- | --- | --- | --- | --- |
| **Pregnancy outcome** | **Pregnancy** | | **Lack of pregnancy** | | **Miscarriage** | |  |  |
| **Before or after IVF-ET** | **before** | **after** | **before** | **after** | **before** | **after** |  |  |
| Number of women | 75 | 74 | 37 | 25 | 37 | 34 | 39 | 27 |
| Minimum | 0.00 | 0.00 | 0.00 | 0.00 | 0.00 | 0.00 | 0.00 | 0.00 |
| 25% Percentile | 211.50 | 431.20 | 0.00 | 0.00 | 76.99 | 94.97 | 910.60 | 311.70 |
| Median | **1134.00^a, b, c^** | **1207.00^d, e, f^** | **0.00^g, h, i^** | **143.30^j, k^** | **414.70^l^** | **721.30^m^** | 1843.00 | 523.80 |
| 75% Percentile | 2017.00 | 2047.00 | 600.80 | 1207.00 | 1409.00 | 1511.00 | 2444.00 | 2100.00 |
| Maximum | 3948.00 | 4123.00 | 3265.00 | 2785.00 | 2368.00 | 2252.00 | 4956.00 | 4575.00 |
| Mean | 1283.00 | 1320.00 | 447.40 | 584.50 | 684.00 | 866.20 | 1850.00 | 1280.00 |
| Std. Deviation | 1052.00 | 1054.00 | 756.70 | 796.00 | 693.40 | 778.00 | 1320.00 | 1419.00 |
| Std. Error | 121.40 | 122.50 | 124.40 | 159.20 | 114.00 | 133.40 | 211.40 | 273.10 |
| Lower 95% CI of mean | 1041.00 | 1076.00 | 195.10 | 256.00 | 452.80 | 594.80 | 1422.00 | 718.50 |
| Upper 95% CI of mean | 1525.00 | 1564.00 | 699.70 | 913.10 | 915.20 | 1138.00 | 2278.00 | 1841.00 |
| D'Agostino & Pearson omnibus normality test K^2^ | 4.46 | 4.52 | 27.25 | 8.54 | 5.57 | 7.72 | 2.59 | 6.59 |
