# Supplementary material for: Pro- and anti-inflammatory cytokines and growth factors in patients undergoing in vitro fertilization procedure treated with prednisone
Source: Front Immunol. 2023 Sep 6;14:1250488. doi: 10.3389/fimmu.2023.1250488 (PMC10511889; doi:10.3389/fimmu.2023.1250488)
Supplement: Supplementary file 11 [file Table_11.docx]

**Supplementary Table 11** VEGF-A value (pg/ml) measured before and after IVF embryo transfer in all patients, including those who received steroid treatment, those who did not and in the fertile controls.

ET – embryo transfer; p values are calculated by Mann-Whitney test:

**Steroid treatment patients before ET vs fertile pregnant control:** ^a^ p = 0.0023;

**Without steroid treatment patients after ET vs fertile pregnant control:** ^b^ p = 0.0102;

**Steroid treatment patients before ET vs fertile control:** ^c^ p = 0.0003;

**Steroid treatment patients before ET vs fertile pregnant control:** ^d^ p < 0.0001;

**Steroid treatment patient after ET vs fertile control:** ^e^ p = 0.0072;

**Steroid treatment patients after ET vs fertile pregnant control:** ^f^ p < 0.0001;

**Fertile control vs fertile pregnant control:** ^e^ p = 0.0244.

| **Study group** | **IVF patients** | | | | **Fertile control** | **Fertile pregnant control** |
| --- | --- | --- | --- | --- | --- | --- |
| **Treatment** | **Without steroid** | | **Steroid** | |  |  |
| **Before or after IVF-ET** | **before** | **after** | **before** | **after** |  |  |
| Number of women | 19 | 13 | 149 | 132 | 38 | 27 |
| Minimum | 0.00 | 5.85 | 0.00 | 0.00 | 0.00 | 0.00 |
| 25% Percentile | 34.20 | 18.72 | 34.47 | 29.03 | 21.29 | 5.70 |
| Median | **58.44^a^** | **67.48^b^** | **62.73^c, d^** | **66.30^e, f^** | **36.80^g^** | 22.62 |
| 75% Percentile | 106.60 | 138.70 | 151.40 | 119.70 | 59.52 | 38.47 |
| Maximum | 448.10 | 392.90 | 809.70 | 764.50 | 134.20 | 82.82 |
| Mean | 88.15 | 95.29 | 116.40 | 103.90 | 45.49 | 28.64 |
| Std. Deviation | 100.90 | 105.10 | 138.70 | 130.10 | 33.41 | 25.81 |
| Std. Error | 23.15 | 29.15 | 11.36 | 11.33 | 5.42 | 4.97 |
| Lower 95% CI of mean | 39.50 | 31.78 | 93.94 | 81.53 | 34.50 | 18.43 |
| Upper 95% CI of mean | 136.80 | 158.80 | 138.80 | 126.40 | 56.47 | 38.85 |
| D'Agostino & Pearson omnibus normality test K^2^ | 29.92 | 16.51 | 104.40 | 94.64 | 11.74 | 3.92 |
