# Supplementary material for: Pro- and anti-inflammatory cytokines and growth factors in patients undergoing in vitro fertilization procedure treated with prednisone
Source: Front Immunol. 2023 Sep 6;14:1250488. doi: 10.3389/fimmu.2023.1250488 (PMC10511889; doi:10.3389/fimmu.2023.1250488)
Supplement: Supplementary file 12 [file Table_12.docx]

**Supplementary Table 12** VEGF-A value (pg/ml) measured before and after IVF embryo transfer in all patients who received steroid treatment, including those who achieved pregnancy, experienced a lack of pregnancy or miscarriage, as well as in the fertile controls.

ET – embryo transfer; p values are calculated by Mann-Whitney test:

**Pregnancy before ET vs lack of pregnancy before ET:** ^a^ p = 0.003;

**Pregnancy before ET vs fertile control:** ^b^ p = 0.0063;

**Pregnancy before ET vs fertile pregnant control:** ^c^ p =< 0.0001;

**Pregnancy after ET vs fertile pregnant control:** ^d^ p = 0.0008;

**Lack of pregnancy before ET vs miscarriage before:** ^e^ p = 0.003;

**Lack of pregnancy before ET vs fertile control:** ^f^ p < 0.0001;

**Lack of pregnancy before ET vs fertile pregnant control:** ^g^ p < 0.0001;

**Lack of pregnancy after ET vs fertile control:** ^h^ p = 0.0084;

**Lack of pregnancy after ET vs fertile pregnant control:** ^i^ p = 0.0005;

**Miscarriage before ET vs fertile control:** ^j^ p = 0.045;

**Miscarriage before ET vs fertile pregnant control:** ^k^ p = 0.0005;

**Miscarriage after ET vs fertile control:** ^l^ p = 0.0073;

**Miscarriage after ET vs fertile pregnant control:** ^m^ p = 0.0003;

**Fertile control vs fertile pregnant control:** ^n^ p = 0.0244.

| **Study group** | **IVF steroid treatment patients** | | | | | | **Fertile control** | **Fertile pregnant control** |
| --- | --- | --- | --- | --- | --- | --- | --- | --- |
| **Pregnancy outcome** | **Pregnancy** | | **Lack of pregnancy** | | **Miscarriage** | |  |  |
| **Before or after IVF-ET** | **before** | **after** | **before** | **after** | **before** | **after** |  |  |
| Number of women | 75 | 74 | 36 | 24 | 38 | 34 | 38 | 27 |
| Minimum | 0.00 | 0.00 | 3.83 | 0.00 | 0.00 | 0.00 | 0.00 | 0.00 |
| 25% Percentile | 31.44 | 25.33 | 47.81 | 32.67 | 31.72 | 39.55 | 21.29 | 5.70 |
| Median | **57.19^a, b, c^** | **57.74^d^** | **143.10^e, f, g^** | **78.41^h, i^** | **52.72^j, k^** | **70.29^l, m^** | **36.80^n^** | 22.62 |
| 75% Percentile | 113.00 | 109.20 | 243.50 | 184.30 | 99.48 | 125.70 | 59.52 | 38.47 |
| Maximum | 792.80 | 764.50 | 809.70 | 650.30 | 409.70 | 427.10 | 134.20 | 82.82 |
| Mean | 100.00 | 96.60 | 182.50 | 129.50 | 86.18 | 102.00 | 45.49 | 28.64 |
| Std. Deviation | 126.80 | 134.30 | 177.80 | 148.90 | 95.40 | 106.10 | 33.41 | 25.81 |
| Std. Error | 14.64 | 15.61 | 29.63 | 30.40 | 15.48 | 18.19 | 5.42 | 4.97 |
| Lower 95% CI of mean | 70.86 | 65.49 | 122.40 | 66.62 | 54.82 | 65.01 | 34.50 | 18.43 |
| Upper 95% CI of mean | 129.20 | 127.70 | 242.70 | 192.40 | 117.50 | 139.00 | 56.47 | 38.85 |
| D'Agostino & Pearson omnibus normality test K^2^ | 75.64 | 71.10 | 26.10 | 25.06 | 25.81 | 21.35 | 11.74 | 3.92 |
